# A Novel Microfluidic System for 3D Epidermis and Full‐Thickness Skin Growth for Nanoparticle Safety Assessment

**DOI:** 10.1002/adhm.202502518

**Published:** 2025-11-02

**Authors:** Samantha Costa, Ana B. Carneiro, Filipa Lebre, João Meneses, Alar Ainla, Cacilda Moura, Ernesto Alfaro‐Moreno, Ana R. Ribeiro

**Affiliations:** ^1^ BiotechHealth PhD Candidate Institute for Biomedical Sciences Abel Salazar of the University of Porto (ICBAS‐UP) Porto 4050‐313 Portugal; ^2^ Master in Biophysics and Bionanosystems School of Sciences of the University of Minho (ECUM) Braga 4710‐057 Portugal; ^3^ International Iberian Nanotechnology Laboratory (INL) Braga 4715‐330 Portugal; ^4^ Department of Bioengineering Technologies Faculty of Science and Technology TechMedCentre University of Twente Enschede 7522 NB The Netherlands; ^5^ Physics Center of Minho and Porto Universities (CF‐UM‐UP) Braga 4710‐057 Portugal

**Keywords:** microfluidics, nanotechnology, nanotoxicology, skin‐on‐chip, titanium dioxide nanoparticles

## Abstract

Chronic skin exposure to nanoparticles (NPs) from air pollution, cosmetics, tattoo inks, and smart textiles is linked to adverse effects such as accelerated aging, dermatitis, eczema, and increased melanoma risk. However, the limited predictive power and physiological relevance of conventional in vitro models, combined with the absence of standardized protocols for assessing NP toxicity, remain a major challenge. To address these limitations, the development of skin‐on‐chip (SoC) systems provides a more physiologically relevant solution, surpassing the constraints of static skin cultures. Here, a novel SoC model with dynamic perfusion and a modular architecture suitable for epidermis‐only (EoC) and full‐thickness (FT) skin models isdeveloped. Under dynamic conditions, both models are metabolically active, exhibit enhanced barrier function, and display a morphology resembling native human skin. Exposure to titanium dioxide (TiO_2_) NPs led to a 32.4% decrease in barrier integrity, a 12.1% reduction in metabolic activity, a 2.9% increase in permeability, and histological evidence of tissue damage. These alterations are associated with an early moderate inflammatory response, as indicated by the upregulation of chemokines. Collectively, these findings demonstrate that the microfluidic device functions as a versatile toxicological tool, with the biological complexity of the FT SoC enhancing its sensitivity for nanotoxicology studies.

## Graphical abstract

This work presents a novel, dynamically perfused, configurable microfluidic system for epidermis‐only (E and full‐thickness skin (FT SoC) growth, emulating human skin structure and barrier function. Upon TiO_2_ nanoparticle exposure, the system reveals compromised barrier integrity, reduced metabolic activity, increased permeability, and chemokine‐driven immune signaling, positioning it as a predictive nanotoxicology tool.

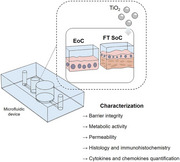

## 1 Introduction

Skin exposure to NPs occurs continuously, intentionally or unintentionally, through airborne nano‐sized particles derived from environmental pollutants, cosmetics and personal care products, intelligent textiles, and tattoos. While the precise mechanisms of action remain largely unknown, there is strong evidence that long‐term exposure to NPs can lead to chronic toxicity, contributing to various skin pathologies, including irritation, dermatitis, sensitization, phototoxicity, eczema, and melanoma.^[^ ^1^ ^]^

The skin's complex architecture, comprising the hypodermis, dermis, and stratified epidermis, plays a crucial role in barrier function and immune surveillance. Key cell types such as keratinocytes, fibroblasts, and immune cells coordinate to maintain skin homeostasis and respond to external stimuli.^[^ ^2^ ^]^ Understanding how NPs interact with these layers requires advanced models capable of mimicking both structural organization and skin physiological function.

Despite the growing prevalence of NP‐containing products, standardized and reliable models for assessing NP toxicity remain lacking. Although several types of in vitro and ex vivo skin models have been developed to study skin physiology and toxicity, they exhibit essential limitations that compromise their predictive value.^[^ ^3^ ^]^ In vitro approaches range from 2D cultures of keratinocytes and fibroblasts to more complex 3D reconstructed human epidermis and FT skin equivalents. Commercial models such as EpiDerm (MatTek), SkinEthic (L'Oréal), LabSkin (Innovenn), and Phenion FT (Henkel) have been widely adopted and validated for regulatory purposes, including irritation, corrosion, and phototoxicity testing.^[^ ^4^, ^5^, ^6^, ^7^ ^]^ These reconstructed models replicate aspects of skin architecture and some barrier functions, but they lack key physiological features, such as vascularization, immune cells, and dynamic perfusion. Their static nature results in uneven nutrient distribution, waste accumulation, and absence of physiological shear stress, all of which can affect cell viability, differentiation, and inflammatory responses over time.^[^ ^8^, ^9^, ^10^ ^]^ Ex vivo human skin explants offer an alternative, retaining the native skin architecture and extracellular matrix (ECM). However, they present limitations such as restricted viability and inter‐donor variability, which limit their use in mechanistic research, safety, and efficacy studies.^[^ ^11^ ^]^ Furthermore, both static in vitro and ex vivo models fail to replicate the dynamic microenvironment of human skin, limiting their ability to assess complex interactions such as NP penetration, accumulation, and inflammatory signaling.^[^ ^12^ ^]^ Consequently, there is an unmet need for advanced in vitro models that more accurately mimic physiological conditions by integrating dynamic perfusion, tissue compartmentalization, and functional readouts, while ensuring both human relevance and reproducibility.^[^ ^13^ ^]^

Organ‐on‐chip (OoC) technology has emerged as a promising alternative to conventional models by enabling dynamic culture conditions, continuous perfusion, and integration of multiple tissue compartments.^[^ ^14^, ^15^ ^]^ Several SoC models have been reported in recent years, including models that incorporate vascular‐like networks, simulate inflammation and edema, or aim to reproduce the microenvironments of melanoma.^[^ ^16^, ^17^, ^18^ ^]^ However, their use in NP toxicity assessment remains limited, with no standardized or validated approaches established. This gap, combined with ethical concerns, the 3Rs principle, and regulations such as the EU ban on animal testing for cosmetics, drives demand for reliable in vitro models for NP safety evaluation.^[^ ^19^ ^]^ Notably, the FDA Modernization Act 2.0 (2022) further supports this shift, allowing validated non‐animal methods like OoC for drug safety testing.^[^ ^20^ ^]^

To address this, a novel microfluidic system with dynamic perfusion and a modular structure was developed for the growth of the EoC and FT SoC. This model introduced a perfusion architecture that accurately recreates the cutaneous microenvironment, enhancing its predictive value for nanotoxicology. Air exposure and ambient temperature were implemented via the epidermal side, while core body temperature and medium flow for nutrient supply were implemented via the dermal side. A removable in‐house transepithelial electrical resistance (TEER) system was integrated into the chip to monitor skin barrier function over time. Metabolic activity, permeability, and skin differentiation markers (keratin 14 – K14, keratin 10 – K10, and loricrin – LOR) were evaluated by PrestoBlue, Lucifer Yellow, and immunohistochemical analyses in both dynamic and static models. As a proof of concept, both the EoC and the FT SoC were exposed to TiO_2_ NPs, and their effect on both models was investigated. This novel microfluidic system offers a dynamic, perfused, and compartmentalized human skin model, enabling continuous nutrient supply, waste removal, and periodic monitoring of barrier integrity. Compared with the literature, our device offers a robust and modular approach that supports both epidermis‐only and FT configurations within a single platform with scalable and reproducible manufacturing. The integration of a removable TEER system allows non‐invasive, longitudinal assessment of barrier function, which is particularly valuable for applications in nanotoxicology. By addressing key limitations of static cultures and providing enhanced functional assessment capabilities, this platform contributes a meaningful advancement toward more predictive, human‐relevant in vitro skin models for NP safety assessment.

## 2 Results and Discussion

### 2.1 Design and Fabrication of the Microfluidic Device

**Figure 1:**
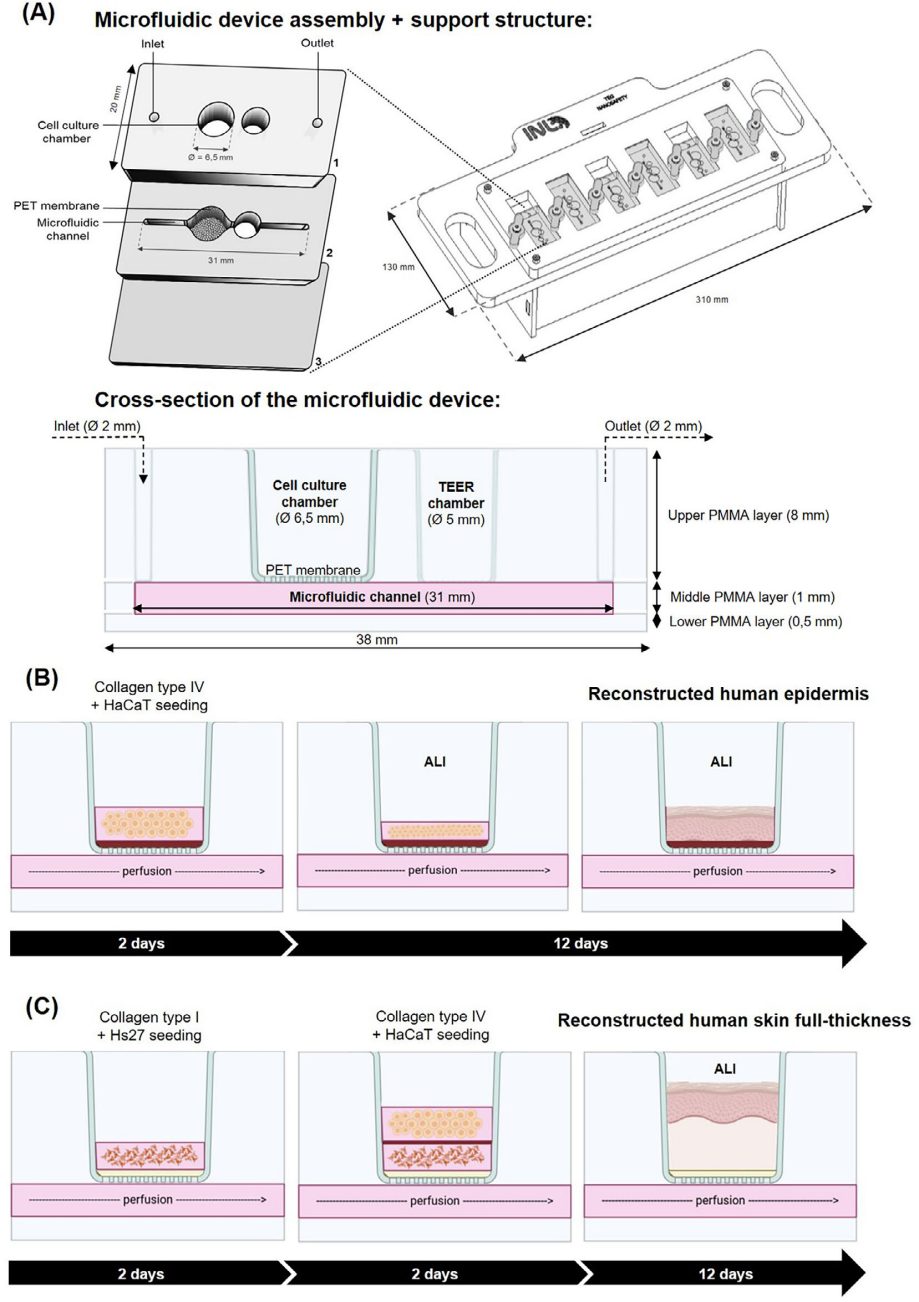
A) Schematic representation of the microfluidic device assembly; the right‐hand schematic shows the arrayed support structure designed to hold six individual devices. A cross‐sectional view of the microfluidic device with all dimensions highlighted is also provided. B) Illustrative diagram of the epidermis reconstruction. Initially, a type IV collagen coating is applied, and the keratinocytes (HaCaT) are seeded. After two days, the system is transitioned to ALI, and TEER measurements are taken every two days. By day 12 in ALI, a reconstructed epidermis is achieved, demonstrating the formation of a structured epidermal layer. C) Illustrative diagram of the skin FT reconstruction. First, a type I collagen coating is applied, followed by the seeding of fibroblasts (Hs27). Next, a type IV collagen coating is added, and the keratinocytes (HaCaT) are seeded. After two days, the system is placed in ALI, and TEER measurements are made every two days. By day 12 in ALI, the FT‐reconstructed skin is formed, indicating the development of structured epidermal and dermal layers.

A novel microfluidic SoC was designed to replicate the physiology of the epidermis and the dermis (**Figure** **1**A). This device, comprising three assembled poly(methyl methacrylate) (PMMA) layers, measures 2.0 cm wide and 3.8 cm long. The upper layer, with a thickness of 8.0 mm, features a central cell culture chamber, as well as a smaller chamber allowing for TEER measurements, with diameters of 6.5 and 5.0 mm, respectively. The cell culture chamber is open at the top, allowing for the seeding of keratinocyte suspensions and collagen gel with fibroblast cells, and enabling air‐liquid interface (ALI) conditions during culture. It was designed to contain an adequate number of cells for accurate and reproducible results. Preliminary experiments established the optimal cell density necessary for reliable toxicological analyses using conventional methods.^[^ ^21^, ^22^ ^]^ The middle layer consists of a microfluidic channel that measures 31.0 mm in length and 1.0 mm in both width and height, ensuring nutrient and oxygen exchange to support long‐term skin culture viability. Furthermore, a 1.0 µm polyethylene terephthalate (PET) porous membrane was sandwiched between the top and the middle layers, separating the cell culture chamber of the microfluidic channel, and the lower layer, with a thickness of 0.5 mm, seals the microfluidic channel. Lastly, the fluidic inlet and outlet have diameters of 2.0 mm. Additionally, a support structure, with a length of 310.0 mm and a width of 130.0 mm, was also designed to hold six chips simultaneously, and its upper part is detachable, allowing for microscopic visualization of the devices.

The fabricated microfluidic device was tested for tightness at a constant flow rate of 1 µL min^−1^, and no water loss was observed, confirming the stability of the chips. This model enabled the reconstruction of the epidermis (Figure 1B) and FT skin (Figure 1C). Overall, the developed model enables in situ biochemical integrity/permeability tests and functional responses to NP exposure, which can be conducted directly on the chips.

#### 2.1.1 Fluid Flow Simulations and In Situ Measurements of Skin Barrier Formation

**Figure 2:**
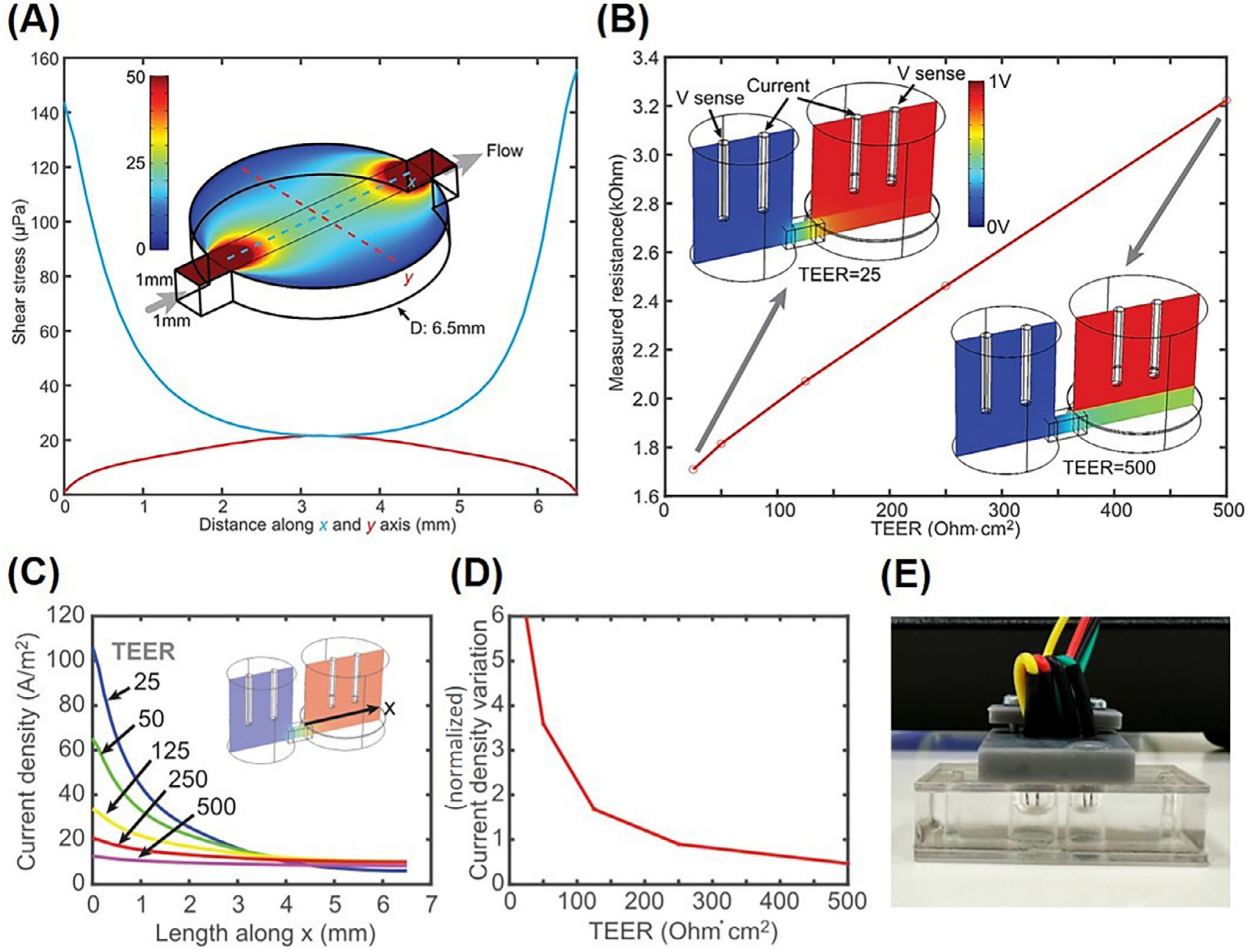
Fluid flow simulations and in situ measurement of skin barrier formation: A) Average shear stress exerted on the chamber walls by the perfusion flow. The inset shows the geometry of the perfusion chamber with color indicating the shear stress. The graph shows the shear stress distribution along the chamber: the x‐axis (blue) between the inlet and outlet, and the y‐axis (red) across the chamber. B) Expected measured resistance depends on the TEER value. The inset figures show simulation geometry, location of electrodes, and potential distribution (0 to 1 V) across the chambers in the case of TEER 25.0 and 500.0 Ohms cm^2^. C) Current density distribution across the membrane along the x‐axis for different TEER values. D) Current density variation across the membrane depends on the TEER value. Variation is defined here as the difference between maximum and minimum current densities divided by the average current density (normalized). E) Photograph of the TEER measurement inset with the four‐electrode configuration used with the developed SoC model.

Fluid flow simulation showed that the maximum shear stress experienced by the cells near the inlet and outlet of the chamber is ≈150.0 µPa (**Figure** **2**A). This result considers a perfectly smooth cell layer. Roughness (e.g., stacked cells) can result in a several‐fold increase in the local shear stress applied to any cell protruding out of the layer toward the center of the chamber. However, even then, the shear stress would be well below the threshold that can be considered harmful for the cells, which is ≈600.0 mPa, 3750 times higher than the highest average shear stress in the chamber.^[^ ^23^ ^]^ To corroborate the fluid flow simulation, Particle Image Velocimetry (PIV) measurements were performed, yielding a maximum shear stress value of 155 µPa (see Figure S1, Supporting Information). This close agreement between simulation and experimental data confirms that the microfluidic environment operates under gentle flow conditions, ensuring cell viability and maintaining the integrity of the skin barrier model.

In turn, finite element modelling (FEM) was used to establish the electrical equivalence of TEER measurements performed on the chip compared to those in traditional well plates. Due to the small channels and substantially different geometries in the chip, the microfluidic circuitry itself can significantly affect the measured electrical resistances. The objective of these simulations was thus to evaluate the effect of the chip on electrical measurements and to support the processing and interpretation of the experimental results. Most importantly, determine the relationship between the measured resistance and the TEER, and also evaluate the current distribution across the cell layer, which describes the variation in the contribution of different tissue regions. For these purposes, the cell layer was considered electrically homogeneous. TEER measurement on the chip is conceptually illustrated in Figure S2 (Supporting Information).

The simulated measured resistance ranges from 1.7 to 3.2 kOhms for TEER values of 25.0 and 500.0 Ohms cm^2^, respectively. While the relationship appears nearly linear, it is important to note that the intercept is not zero. In the absence of a membrane, the measured resistance would be ≈1.5 kOhms, which accounts for almost half of the total resistance. These values are primarily due to the intrinsic contribution of the chip itself. As can be seen in Figure 2B, the main voltage drop–and thus the primary source of resistance–occurs at the interconnection between the two chambers. However, the lower perfusion channel also contributes significantly to the total resistance. This contribution from the microfluidic channel is one of the reasons why the current distribution is not homogeneous, particularly in cases with lower TEER values.

Figure 2C shows the current density profile along the membrane, where the current is the highest near the inlet channel. The difference becomes proportionately higher when TEER is lower (Figure 2D). In the case of TEER = 25.0 Ohms cm^2^, the current density difference between maximum and minimum over the average current density can be as high as six‐fold, meaning cells near the inlet influence the average 6 times more than at the opposing corner. In the case of homogeneous cell layers, this difference is, however, not important.

Finally, to minimize the impact from electrodes and drifts, it is beneficial to use a four‐electrode configuration (Figure 2E) rather than a two‐electrode configuration, in which case current‐driving working electrodes and potential‐sensing reference electrodes are separated, eliminating current‐dependent potential drops on the working electrodes.

The difference between the medium near the working electrode and the average potential on the reference electrodes was evaluated. The difference was small, between 16.0 and 24.0 mV per electrode in the case of TEER 500.0 and 25.0 Ohms cm^2^, respectively. Even though this is a systematic effect and can be compensated by calculation, even if not considered, the maximum resistance error would be only 1.8% in the full range. These results from the simulation could be used to interpret the measurements using the TEER sensing feature in the developed SoC model.

### 2.2 Human Fibroblasts and Keratinocytes Characterization

HaCaT and Hs27 cell lines have been increasingly used in SoC research due to their relevance in modelling epidermal and dermal layers.^[^ ^24^, ^25^, ^26^ ^]^ Studies by Wufuer et al. and Jeon et al. notably employed both cell lines together.^[^ ^17^, ^27^ ^]^ Phase contrast microscopy images (Figure S3A, Supporting Information) taken throughout subculturing in T75 flasks show that HaCaT cells maintain a consistent cuboidal morphology, while Hs27 cells are large, flat, and elongated (spindle‐shaped) with oval nuclei. Both cell lines have a similar doubling time of ≈24 to 28 h. The Live/Dead assay (Figure S3B, Supporting Information) results at 24, 48, and 72 h post‐seeding confirm high viability and rapid proliferation. Additionally, both cell lines were authenticated and confirmed to be free of mycoplasma contamination, ensuring reliable and reproducible experimental outcomes. The HaCaT and Hs27 cell lines used are widely characterized in the literature, and their stable morphology and proliferation behavior across passages support their relevance for 3D skin model development.

### 2.3 Generation and Functional Validation of the Epidermis‐On‐Chip

The establishment of the epidermal layer structure and its barrier function primarily occurs through the proliferation of keratinocytes, where the ALI step is crucial for the development of an epidermal model, as it allows differentiation of the keratinocytes.^[^ ^28^ ^]^ In the developed EoC model, HaCaT keratinocyte cells were seeded on the central, open‐top chamber of the chip, which was pre‐coated with collagen type IV, an essential component of the ECM, to form a stable epidermal‐like layer. The ECM provides a specific niche that mediates mechanical and chemical signals to keratinocytes through cell‐ECM interactions.^[^ ^29^ ^]^

**Figure 3:**
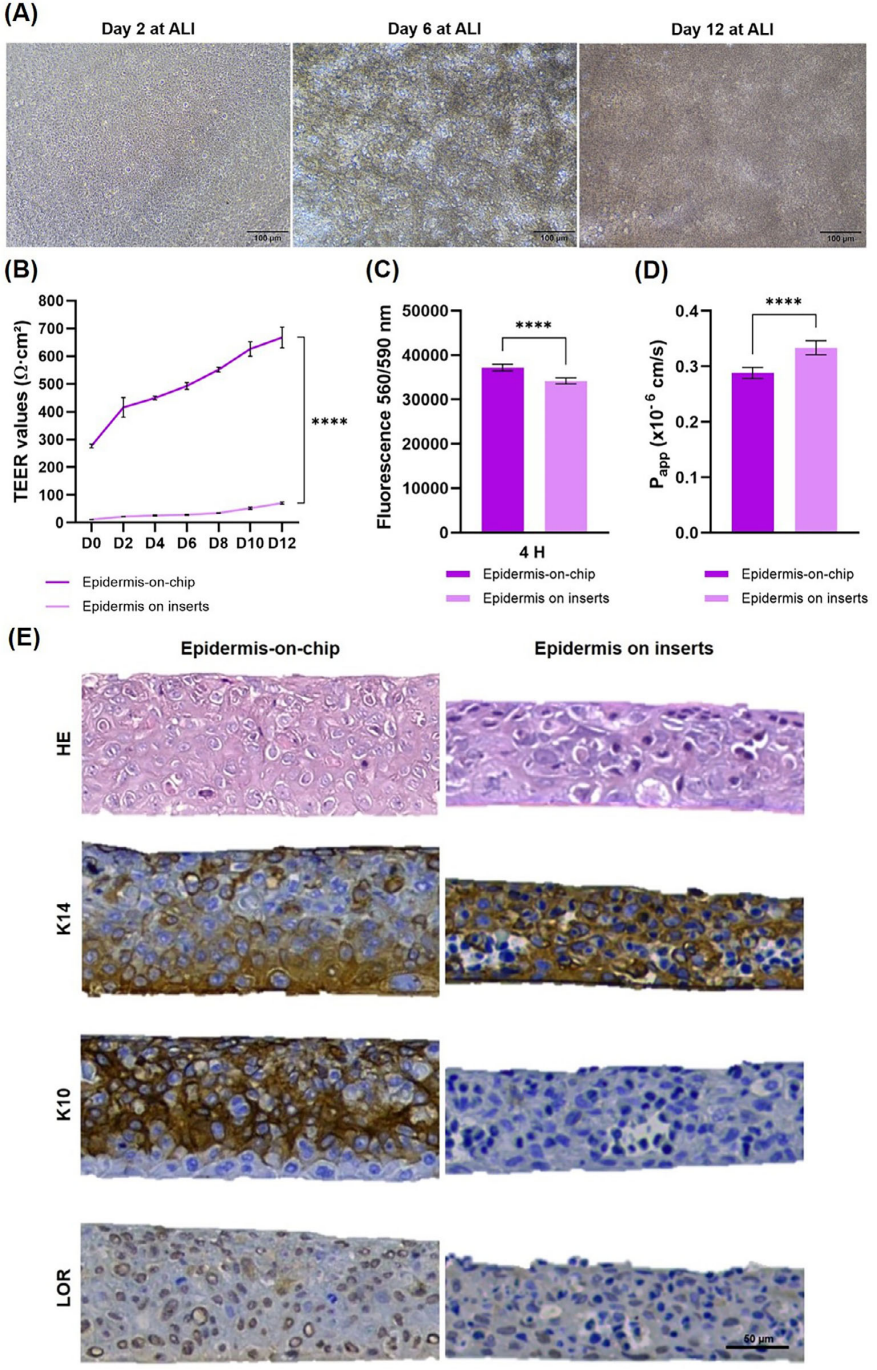
Epidermis‐on‐chip characterization: A) Phase contrast images showing the differentiation of HaCaT keratinocyte cells on the EoC after 2, 6, and 12 days at ALI. B) Barrier integrity measured by TEER from day 0 to day 12 at ALI. C) Metabolic activity assessed through fluorescence measurements using PrestoBlue^TM^. D) Apparent permeability coefficient determined by Lucifer Yellow fluorescence. E) Epidermis morphology and biomarkers expression: HE staining showing overall tissue morphology and immunohistochemistry for K14, K10, and LOR expression. Quantitative data B–D) are presented as mean ± SD, with n = 8 independent assays, each performed with six technical replicates (NS *p* > 0.05, *p < 0.05, **p < 0.01, ***p < 0.001, and ****p < 0.0001).

Forming a layer of keratinocyte cells that covers the entire membrane over time (**Figure** **3**A) is essential to assess the integrity, viability, and permeability characteristics of the skin. HaCaT keratinocyte cells were successfully cultured on the PET membrane (1.0 µm pore size), demonstrating the feasibility of the proposed cell density and culturing method. However, this cell line does not form a stratum corneum.^[^ ^29^ ^]^ In this context, a specific differentiation culture medium was used to aid in the maturation and differentiation of the keratinocyte cells, containing a combination of growth factors and components, as described in Section Reconstructed Human Epidermis and Full‐Thickness Skin‐on‐Chip:Epidermis‐On‐Chip. Font et al. showed that the use of Dulbecco's Modified Eagle Medium (DMEM) and Ham's‐F12 Nutrient Mixture significantly enhances cell differentiation, resulting in a simplified reconstructed epidermis whose architecture is similar to the in vivo epidermis.^[^ ^30^ ^]^ Furthermore, apo‐transferrin, insulin, and transforming growth factor beta (TGF‐β) promote cell growth and multiplication, while epidermal growth factor (EGF) induces proliferation and reduces apoptosis.^[^ ^31^, ^32^ ^]^ Additionally, hydrocortisone acts as a potent stimulator of barrier properties.^[^ ^33^ ^]^ Moreover, fibroblast‐conditioned medium secretes growth factors and ECM molecules, serving as an effective natural healing agent that facilitates keratinocyte expansion.^[^ ^34^ ^]^ The successful differentiation of keratinocytes is essential for enhancing the physiological relevance of the EoC model despite the inherent limitations of the HaCaT cell line.

To quantitatively evaluate the skin barrier function in EoC, a custom reusable electrode holder for the chip was designed, allowing precise and consistent placement of four electrodes into the chambers of different chips (Figure S4, Supporting Information). Monitoring of TEER values (every two days from day 0 at ALI) showed a consistent increase, indicating the progression of the culture in ALI and enhancement of the epidermal barrier function. Higher TEER values are indicative of more robust tight junctions, further confirming the strengthening of the skin barrier. Furthermore, it was demonstrated that perfusion‐based cell culture promotes an improved barrier on the EoC, as the model presents a significantly higher mean TEER (667.95 ± 37.95 Ohms cm^2^) compared to the static control (71.10 ± 4.02 Ohms cm^2^). This improvement may be attributed to the continuous replenishing of cell nutrients and the removal of cellular waste, which may likely contribute to the stable growth of cells and improved differentiation, leading to long‐term cell survival.^[^ ^35^ ^]^ However, as shown in Figure 3B, the TEER values measured are relatively low, which is consistent with the literature for this cell line.^[^ ^36^ ^]^ HaCaT cells, despite reaching confluence, do not form tight junctions as effectively as primary keratinocytes, resulting in lower resistance values.^[^ ^37^ ^]^

A key aspect of the successful operation of the EoC model is the efficient transport of nutrients from the microfluidic channel to the epidermal layer of the skin. Skin metabolic activity, related to cell viability, was analyzed using PrestoBlue (Figure 3C). After 12 days of cell culture at ALI, the metabolic activity of the keratinocytes was assessed, confirming that cells remained metabolically active in both the chips and static inserts. Notably, significant differences were observed between them, which can be attributed to several factors. The microfluidic device creates a more controlled and physiologically relevant microenvironment, promoting efficient nutrient diffusion and enhanced removal of toxic metabolites.^[^ ^38^ ^]^ Additionally, the dynamic flow within the chips introduces beneficial shear stress that further supports cell viability.^[^ ^24^ ^]^

Skin permeability studies were also conducted after 12 days of cell culture at ALI, using Lucifer Yellow, a hydrophilic fluorescent molecule commonly employed to assess paracellular transport and thus serve as an indicator of barrier integrity. The apparent permeability coefficient (P_app_) was determined using Fick's first law of diffusion. Remarkably, the EoC model exhibited a significantly lower P_app_ compared to the static inserts (Figure 3D). This confirms that the EoC model exhibits robust barrier function and enhanced barrier integrity, as evidenced by its low permeability and elevated TEER values. Furthermore, increased cellular viability in the EoC model reinforces its capacity to mimic the native barrier properties while maintaining cellular function.

Histological and immunohistochemical analyses (Figure 3E) revealed no variability among the various reconstructed tissues and confirmed the growth of epidermal cells. According to the literature, cells in the stratum basale have a cuboidal shape. From the second layer on toward the upper layers, keratinocytes differentiate, altering their cell morphology, becoming spinous in the stratum spinosum and flattened in the stratum granulosum, where keratohyalin granules appear as purple dots in the cytoplasm.^[^ ^39^ ^]^ The hematoxylin‐eosin (HE) staining demonstrates these morphological changes across the basal, squamous, and granular layers. Additionally, the expression of K14 in the basal layer and K10 in the squamous layer further confirms the identification of these layers. However, the reduced presence of brown‐staining for LOR indicates the limited formation of the stratum corneum, which is consistent with our expectations for this model.^[^ ^40^ ^]^ Moreover, the epidermal layer on the chip is thicker than in the static model. This increased thickness can be attributed to the dynamic culture conditions in the chip, which better mimic the physiological environment, promoting enhanced cell proliferation and differentiation.

In summary, the results of epidermal barrier integrity, cellular metabolic activity, permeability, and skin differentiation markers demonstrate that the developed microfluidic device enables the growth of a stable and confluent epidermis, highlighting the device's stability and biocompatibility. It is also important to note that although the absolute TEER values observed in this model remain relatively low due to the inherent characteristics of the HaCaT cell line, the substantial reduction in P_app_ demonstrates that the overall barrier function is effectively established. This reinforces that TEER and P_app_ measure different but complementary aspects of barrier integrity: TEER predominantly reflects ionic conductance through tight junctions, whereas P_app_ quantifies the paracellular transport of small fluorescent tracer molecules across the epidermal layer. This transport occurs primarily through intercellular spaces and is influenced by factors such as tight junction integrity, tissue compactness, and cellular viability.^[^ ^41^, ^42^ ^]^ Together, TEER and P_app_ offer a comprehensive assessment of the barrier's structural and functional properties.

### 2.4 Establishment and Characterization of the Full‐Thickness Skin‐On‐Chip

**Figure 4:**
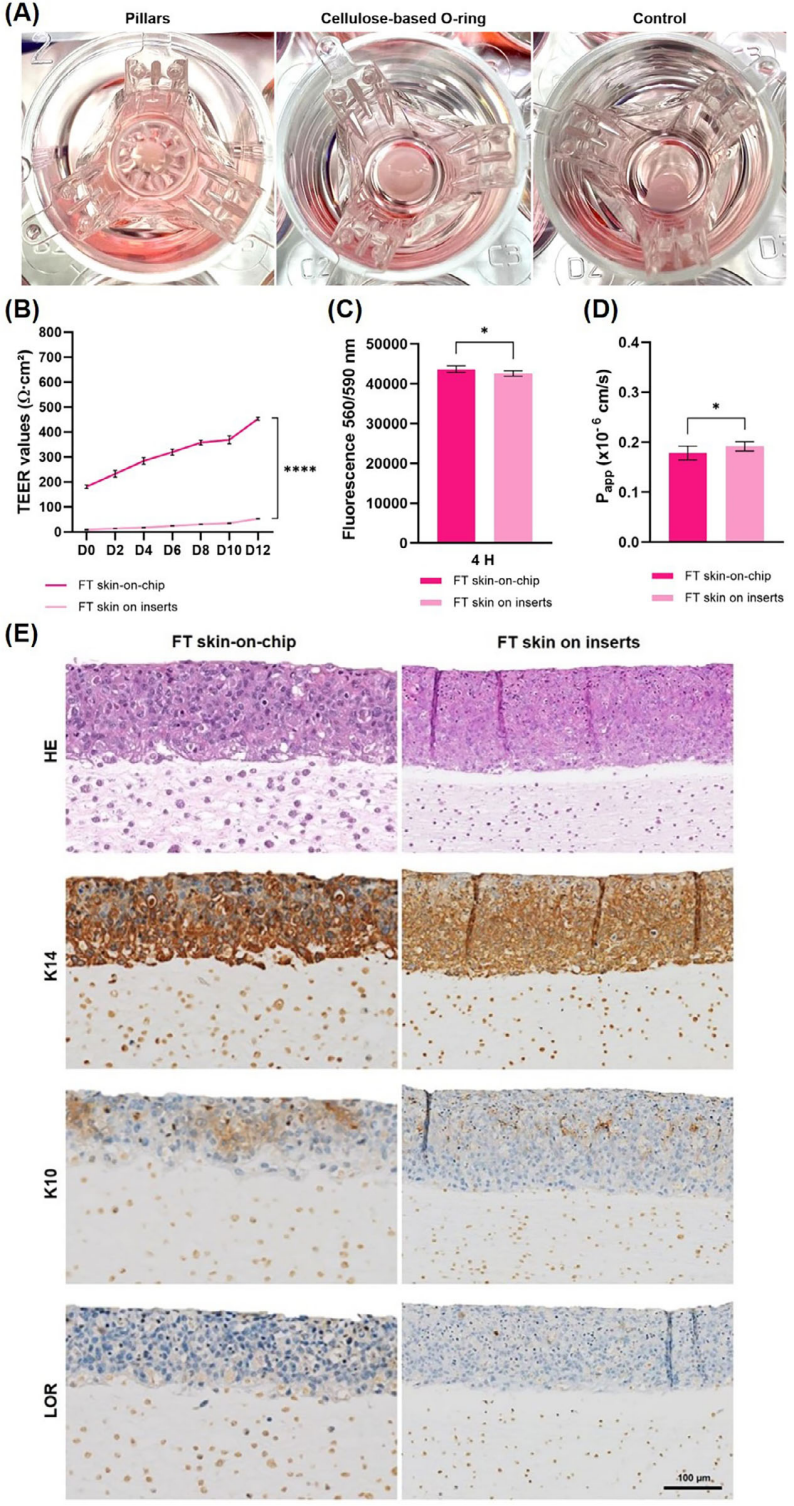
Full‐thickness skin‐on‐chip characterization: A) Collagen contraction assay in static conditions, using a collagen type I concentration of 6.0 mg cm^−3^ and a volume of 200 µL evaluated in pillars, cellulose‐based O‐ring, and control conditions. B) Barrier integrity assessment via TEER measurements from day 0 to day 12 at ALI. C) Metabolic activity quantified by fluorescence using PrestoBlue. D) Apparent permeability coefficient determined by Lucifer Yellow fluorescence. E) Skin morphology and biomarkers expression: HE staining showing overall tissue morphology and immunohistochemistry for K14, K10, and LOR expression to assess epidermal stratification and differentiation. Quantitative data B–D) are presented as mean ± SD, with n = 8 independent assays, each performed with six technical replicates (NS *p* >0.05, *p < 0.05, **p < 0.01, ***p < 0.001, and ****p < 0.0001).

While collagen remains a common choice for constructing in vitro skin dermis models, it does come with certain drawbacks. One significant disadvantage is the contraction of the collagen matrix induced by fibroblasts. Through their interaction with the ECM, fibroblasts exert traction forces that reorganize collagen fibers into denser structures, leading to a reduction in matrix volume and deformation of the dermal layer. This excessive matrix contraction in the collagen matrix can compromise the skin dermis model's functionality.^[^ ^43^ ^]^ To prevent collagen type I contraction into the designed FT SoC, two collagen concentrations – 3.0 and 6.0 mg cm^−3^ ‐ and different volumes – 100, 200, and 300 µL – were used, combined with two physical approaches: i) the implementation of pillars, and ii) the use of cellulose‐based O‐rings. Results demonstrate that the optimal conditions that avoid collagen contraction comprise a collagen type I concentration of 6.0 mg cm^−3^ and a collagen volume of 200 µL when using a cellulose‐based O‐ring (**Figure** **4**A). In this context, the cellulose‐based O‐ring serves as a physical constraint, providing lateral mechanical support that confines collagen within a fixed geometry and prevents excessive contraction during fibroblast‐mediated remodeling.

Due to the substantial thickness of the FT skin tissue, visualizing and capturing images using the inverted optical microscope was not feasible. Similarly to the EoC, TEER measurements exhibit a progressive increase over time, reaching 453.05 ± 5.85 Ohms cm^2^ in microfluidic devices and 53.30 ± 1.50 Ohms cm^2^ in static controls, confirming the successful growth of cells and barrier formation (Figure 4B). The difference in TEER values between the FT SoC and the static can be explained by the conductance in the microfluidic channels. An equal current density over the entire membrane is normally required to conduct TEER experiments properly. The bulk liquid in the inserts ensures an approximately uniform potential drop throughout the whole membrane due to the conductivity of the culture medium. This is not always the case in microfluidic chips because the conductivity of microfluidic channels is often significantly lower than in bulk. As a result, current distribution through the uniform membrane can be uneven, leading to higher apparent TEER in chip systems compared to static inserts with the same membrane area.^[^ ^44^ ^]^ Comparably to what was observed in the EoC, the TEER values recorded in the FT SoC are lower than those described in the literature. This can be attributed to the presence of the dermal layer, which is less compact and contains fewer tight junctions, leading to increased ionic permeability and consequently reduced electrical resistance.^[^ ^45^ ^]^ Zoio et al. reported TEER values of 1393 ± 255 Ohms cm^2^ in a SoC model with integrated tetrapolar electrodes, with measurements taken after 11 days at ALI using primary human epidermal keratinocytes, which form tighter junctions and a more robust barrier.^[^ ^46^ ^]^ In another study, Sriram et al. obtained TEER values of 6200 ± 300 Ohms cm^2^ using Ag/AgCl electrodes, with values recorded after 20 days at ALI using immortalized human N/TERT‐1 keratinocytes. The ability of this cell line to form a stratum corneum contributes to the significantly elevated TEER values observed.^[^ ^47^ ^]^

At the end of the human FT skin reconstruction, the metabolic activity was assessed, confirming that cells remained metabolically active in both FT SoC and static controls (Figure 4C). This indicates that cellular viability was maintained across both conditions. Furthermore, a reduction in the P_app_ of the FT SoC (Figure 4D) was observed when comparing the results with the static controls. Statistical analysis revealed a significant difference in metabolic activity and P_app_, suggesting that the cellular viability and barrier function of the FT SoC were superior to those of the static inserts.

Histological and immunohistochemical analyses (Figure 4E) clearly showed the formation of a dermal section and an epidermal stratification, confirming tissue growth after 12 days at ALI. The expression of K14 in the basal layer strongly supports the identification of the basal layer, while only a very faint expression of K10 was observed in the upper layers, indicating the presence of the spinous layer but with limited differentiation. This reduced expression of K10 could be attributed to the thickness of the dermis, which may have impaired the efficient delivery of nutrients to the upper layers of the epidermis, thereby limiting keratinocyte differentiation. Additionally, the limited brown‐staining for LOR further supports the conclusion that the reconstructed FT SoC model does not undergo cornification, similar to the observations in the EoC.

Taken together, all these results underscore the potential of the FT SoC model to support the growth of a skin structure with dermal and epidermal layers mimicking native human skin. It is also worth noting that although the absolute TEER values in the FT SoC remain lower than those reported for primary cell‐based models, the concurrent reduction in P_app_ confirms that the FT SoC establishes an effective barrier. This further supports the view that TEER and P_app_ provide complementary information, reflecting different aspects of skin barrier integrity and functionality.

### 2.5 Safety Assessment of Titanium Dioxide Nanoparticles

#### 2.5.1 Physicochemical Characterization and Optimization of a Dispersion Protocol for Titanium Dioxide Nanoparticles

TiO_2_ NPs, particularly in their rutile form, are widely used in sunscreen formulations due to their effective UV‐blocking properties. However, despite their widespread use, there is ongoing controversy regarding their potential toxicity.^[^ ^48^ ^]^ Given the concerns about their safety, it is essential to investigate their effects in more physiologically relevant in vitro models, such as EoC and FT SoC models. Before exposing these NPs to the developed systems, a thorough characterization of their properties was conducted, including X‐ray diffraction (XRD), Raman spectroscopy, X‐ray photoelectron spectroscopy (XPS), and scanning transmission electron microscopy (STEM) analyses, to confirm their structure and chemical composition. Dynamic light scattering (DLS) was also used to measure the mean particle size and polydispersity index (PDI) of these NPs.

**Figure 5:**
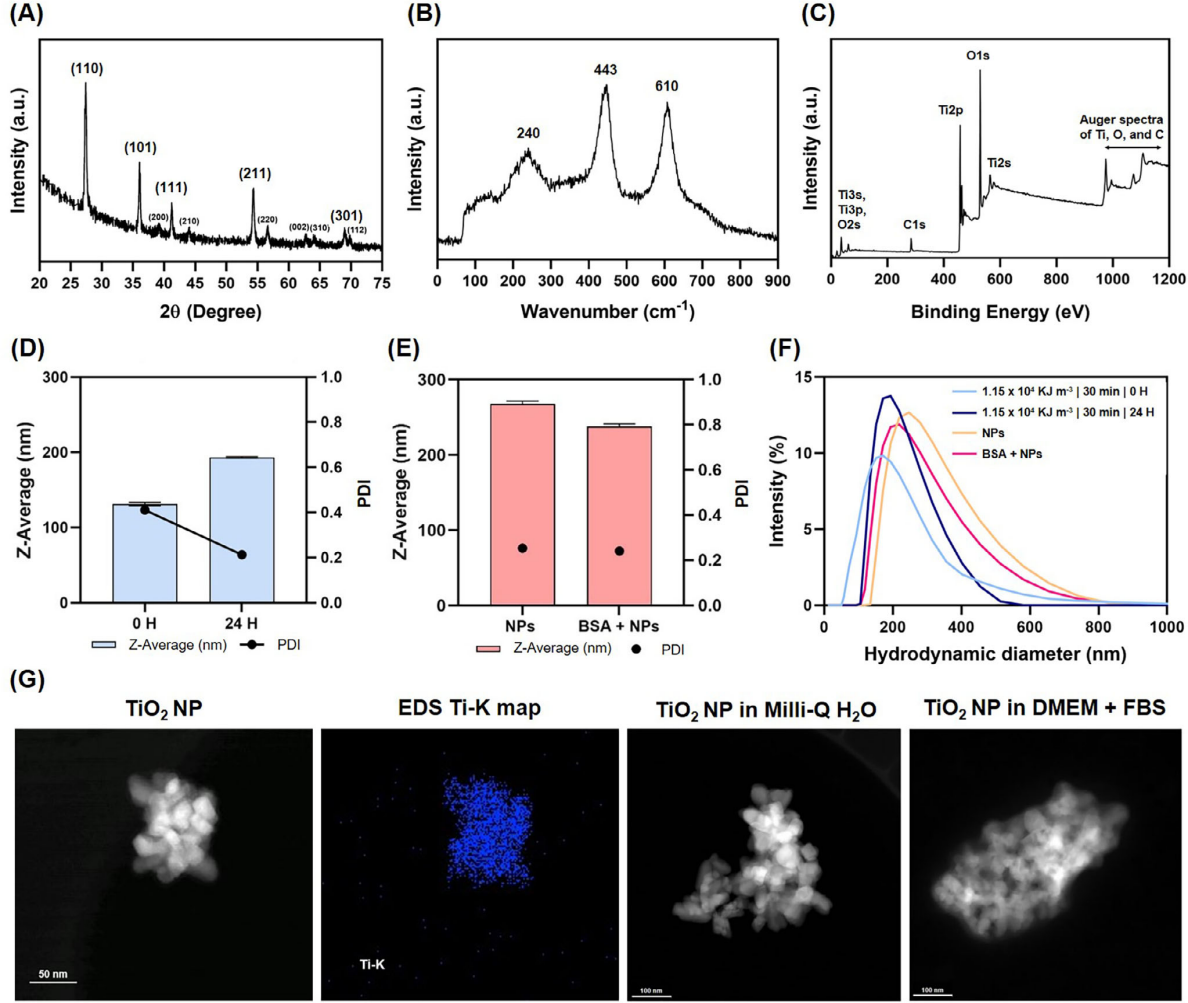
Physicochemical characterization and dispersion optimization of TiO_2_ NPs: A), B), and C) X‐ray diffraction, Raman spectroscopy, and XPS spectra, respectively. D) Z‐average and corresponding PDI, 0 and 24 h after stabilization, of a final concentration of 0.5 mg mL^−1^ TiO_2_ NP suspensions in water, sonicated with an energy of 1.15 × 10^4^ KJ m^−3^ for 30 min. E) Z‐average and corresponding PDI of the dispersed TiO_2_ NPs stock solution stabilized for 24 h, diluted in DMEM + 10% FBS, and in DMEM + 10% FBS with the previous addition of BSA as a stabilizing agent. F) Particle size distribution of each measurement. G) STEM micrographs of: TiO_2_ NPs without dispersion, EDS Ti‐K map confirming titanium presence (in blue), and TiO_2_ NPs agglomerate in Milli‐Q water and complete culture medium.

The rutile crystal structure was confirmed by XRD (**Figure** **5**A). All the diffraction peaks were identified as characteristic of the XRD pattern of rutile TiO_2_. The presence of diffraction peaks at (110), (101), (111), (211), and (301) indicates that it is a crystalline rutile TiO_2_ phase. No additional peaks corresponding to impurities and other structural phases were detected. The Raman spectrum (Figure 5B) exhibited major peaks at 401 and 638 cm^−1^, with a minor peak at 247 cm^−1^, confirming the rutile phase of the NPs. To determine the composition and identify the chemical states of rutile TiO_2_ nanocrystals, XPS analysis (Figure 5C) was carried out. The two strong peaks from the rutile at around 459.5 eV with symmetry can be attributed to Ti2p1/2 and Ti2p3/2. The peak positions and 5.8 eV peak separation of the Ti2p doublet agree well with the energy reported for TiO_2_ NPs. In turn, the O1s peak is located at ≈529.09 eV. Ti2p and O1s peak positions and the line shapes are characteristic of stoichiometric TiO_2_. The carbon source is probably surface contamination with adventitious hydrocarbons.

After the physicochemical characterization, TiO_2_ NPs were suspended in ultrapure water and dispersed using direct probe sonication, resulting in a mean hydrodynamic size of 131.33 ± 2.23 nm at 0 h and 193.40 ± 0.94 nm at 24 h of stabilization, as determined by DLS (Figure 5D). After incubating TiO_2_ NPs in the complete DMEM, the mean hydrodynamic size increased to 267.70 ± 4.00 nm, and the PDI reached 0.254 (Figure 5E). The ionic strength of the culture medium alters the surface charge of NPs, reducing electrostatic repulsion and promoting agglomeration. Additionally, the culture medium is also rich in different proteins present in serum that are known to adsorb onto TiO_2_ NPs.^[^ ^49^ ^]^ To mitigate agglomeration, bovine serum albumin (BSA) was added to the suspension before DMEM incubation, serving as a stabilizing agent.^[^ ^50^ ^]^ A decrease in rutile hydrodynamic size and PDI was observed (hydrodynamic size = 237.37 ± 3.86 nm, and PDI = 0.241). This suggests that the addition of BSA may help prevent excessive agglomeration by modifying the protein corona and stabilizing the particles. Particle size distributions for each measurement are shown in Figure 5F, including those for the dispersion in Milli‐Q water under ideal conditions at 0 and 24 h, as well as for the dispersion in DMEM with 10% fetal bovine serum (FBS) with and without the addition of BSA. STEM micrographs (Figure 5G) reveal the morphology of the NPs with and without dispersion, and the EDS map confirms the presence of titanium.

#### 2.5.2 Effect of Titanium Dioxide Nanoparticles on the Epidermis and Full‐Thickness Skin‐On‐Chip

Once the EoC and FT SoC constructs are fully developed, the tissues can be exposed to various stressors (e.g., NPs), allowing the study of how different NPs may activate mechanisms like oxidative stress or inflammation. During this exposure, the tissues are usually maintained in submerged conditions. In these experiments, TiO_2_ NPs were applied on day 12 at ALI, after the EoC and FT SoC constructs were fully developed.

**Figure 6:**
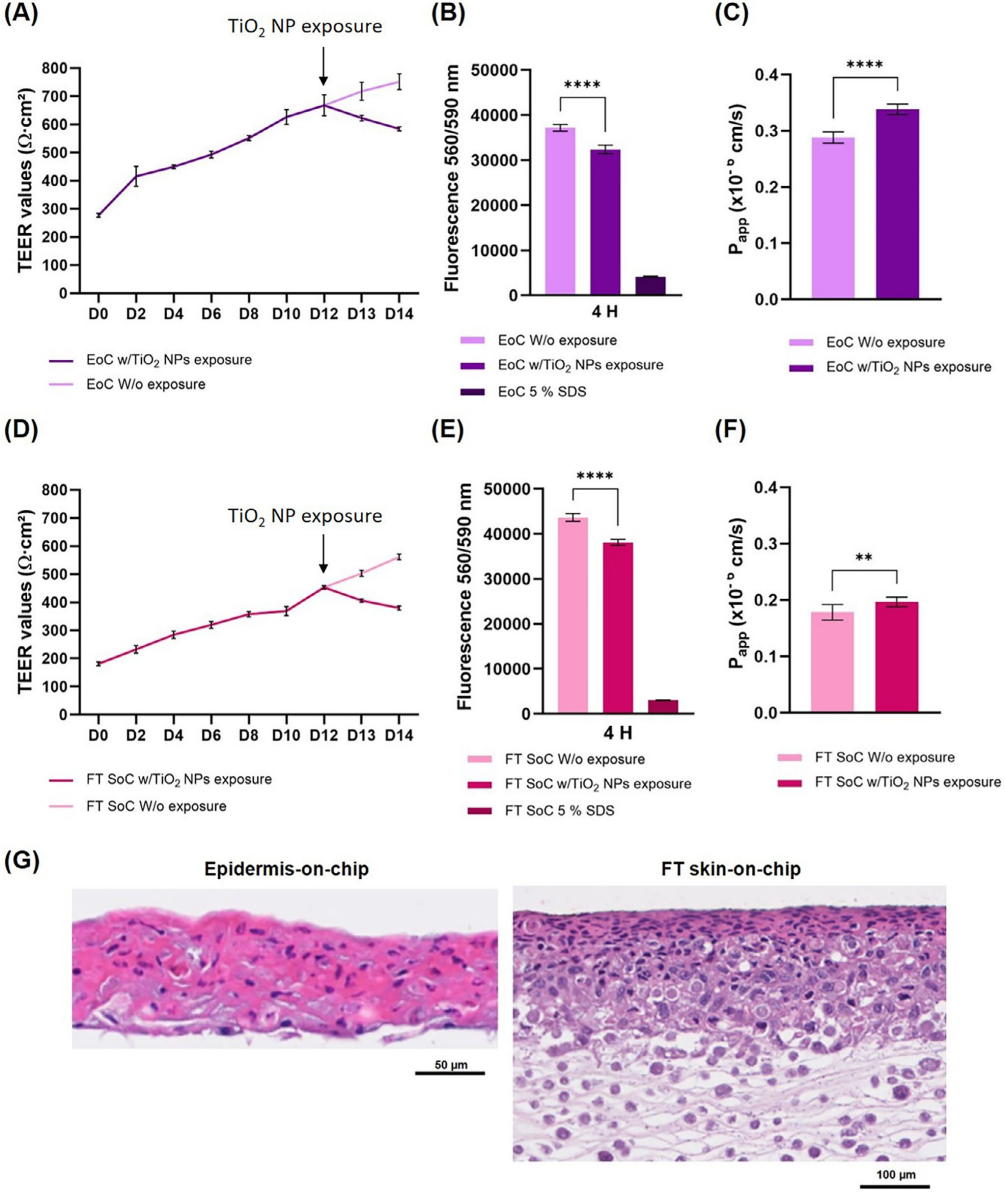
Exposure of TiO_2_ NPs in EoC and FT SoC: A) EoC barrier integrity measured by TEER from day 0 to day 14 at ALI, with TiO_2_ NPs exposure on day 12. B) EoC metabolic activity evaluated by fluorescence using PrestoBlue. C) EoC apparent permeability coefficient determined by Lucifer Yellow fluorescence. D) FT SoC barrier integrity measured by TEER from day 0 to day 14 at ALI, with TiO_2_ NPs exposure on day 12. E) FT SoC metabolic activity evaluated by fluorescence using PrestoBlue. F) FT SoC apparent permeability coefficient determined by Lucifer Yellow fluorescence. G) EoC and FT SoC morphology after TiO_2_ NPs exposure. Quantitative data A–F) are presented as mean ± standard deviation, with n = 8 independent experiments, each performed with six technical replicates (NS *p* >0.05, *p < 0.05, **p < 0.01, ***p < 0.001, and ****p < 0.0001).

Based on the group's previous experience, a specific concentration of ≈33.33 µg cm^−2^ was selected to expose the model to.^[^ ^49^ ^]^ After 48 h of exposure, TEER measurements, metabolic activity, and permeability assays were performed. **Figure** **6**A,D demonstrate a slight decrease in TEER values upon TiO_2_ NPs exposure compared to EoC and FT SoC without NPs exposure. This reduction in TEER can be related to the possible disruption of cellular tight junctions that possibly compromise skin barrier integrity. Alternatively, NPs deposition on the cell surface could also interfere with the barrier function by either physically obstructing the tight junctions or inducing cellular stress, which can also lead to barrier disruption. Figure 6B,E equally show a significant decrease in metabolic activity in EoC and FT SoC with TiO_2_ NPs exposure compared to those without NPs exposure. This decrease in metabolic activity suggests a cytotoxic effect, potentially resulting from oxidative stress or inflammation induced by the TiO_2_ NPs. Most mechanisms of NP‐related skin effects have been attributed to oxidative stress and subsequent inflammatory responses, autophagy, cell membrane damage, and necrosis, with sunlight exposure intensifying the effects.^[^ ^51^ ^]^ Consistently, an increase in the P_app_ of the EoC and FT SoC models exposed to TiO_2_ NPs was observed (Figure 6C,F), further supporting the notion that the barrier integrity was compromised by TiO_2_ NPs exposure. Sodium Dodecyl Sulphate (SDS) 5% was used as a positive control. SDS is recommended by the Organisation for Economic Co‐operation and Development (OECD) and Interagency Coordinating Committee on the Validation of Alternative Methods (ICCVAM) to be used as the reference cytotoxic substance for in vitro tests to assess the toxic effects of substances on skin and the reproducibility of the quantitative response of test systems, based on healthy human keratinocytes, to toxic exposure (test system validation).^[^ ^52^ ^]^ In turn, the histological analyses of the tissues (Figure 6G) showed increased eosinophilic staining in the outermost layer of the tissue in both EoC and FT SoC models, suggesting potential tissue damage.^[^ ^53^ ^]^ This increased eosin staining may be attributed to structural and cellular changes resulting from oxidative stress, inflammatory response, and cellular damage induced by TiO_2_ NPs exposure. These changes could include alterations in cellular morphology, such as swelling or disruption of cellular structures upon NPs internalization, which would lead to greater eosin uptake.^[^ ^54^ ^]^ These findings are consistent with the disturbance of tissue integrity observed in TEER measurements and permeability assays and align with the known ability of NPs to penetrate the skin via multiple pathways, including hair follicles, transcellular and paracellular transports, sweat glands, skin folds, or a combination thereof.^[^ ^48^ ^]^

### 2.6 Cytokines and Chemokines Analysis and Quantification

**Figure 7:**
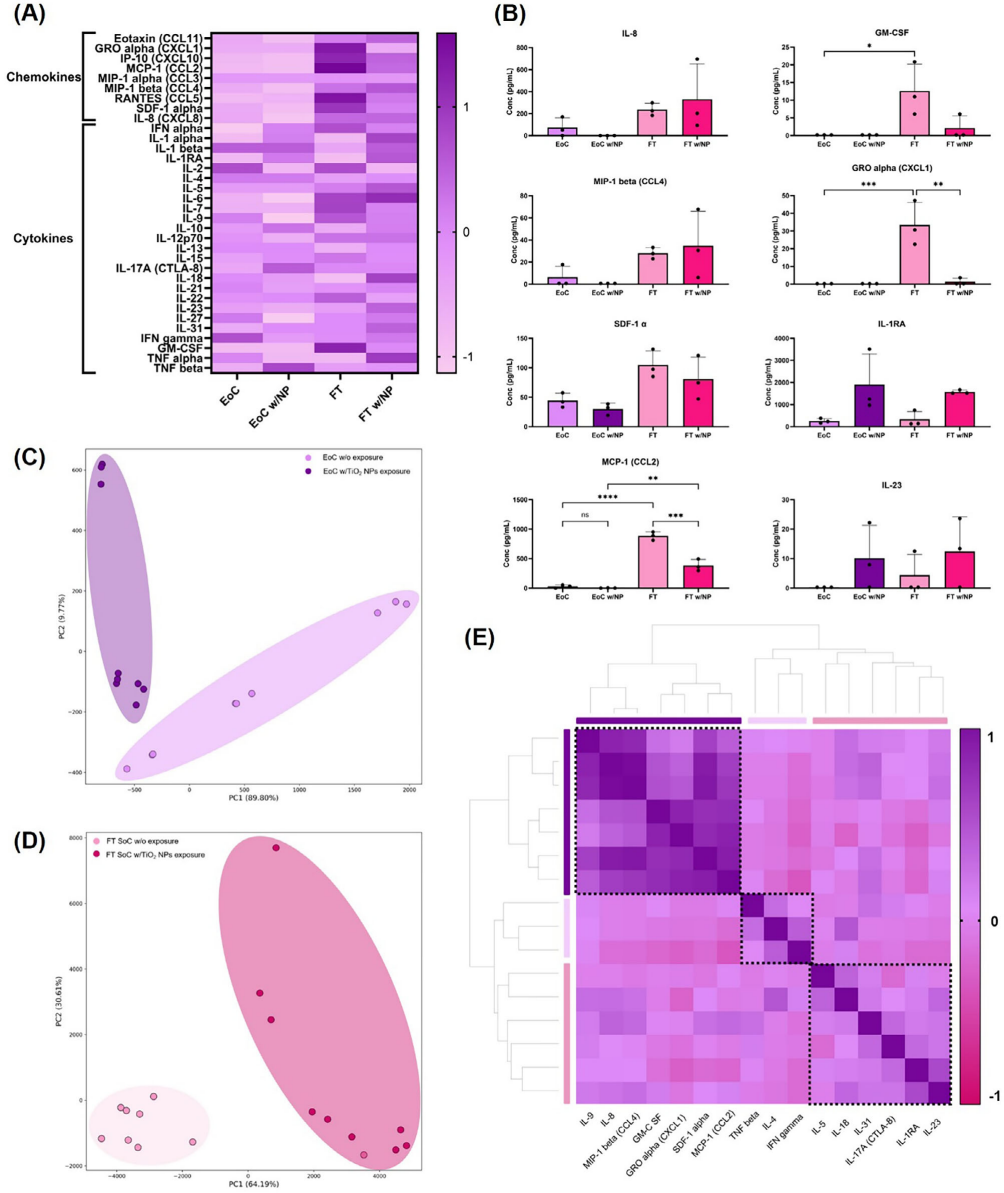
EoC and FT SoC models were exposed to TiO_2_ NPs (33.33 µg cm^−2^) for 48 h. Supernatants were collected before and after exposure and tested by multiplex assay. A) Heat map visualization depicting cytokine and chemokine expression profiles of EoC and FT SoC before and after TiO_2_ NP exposure. Data from 3 independent assays are expressed as a z‐score. B) Cytokine and chemokine levels are presented as mean concentrations ± SD, with n = 3 independent assays, tested in triplicate (NS *p* >0.05, *p < 0.05, **p < 0.01, ***p < 0.001, and ***p < 0.0001). C) PCA plot of all samples collected from the EoC model before and after 48 h of exposure to TiO_2_ NPs. D) PCA plot of all samples collected from the FT model before and after 48 h of exposure to TiO_2_ NPs. E) Cluster map depicting the hierarchical correlation of the different cytokine and chemokine mediators. The map is color‐coded as shown in the legend.

Skin cells continuously produce baseline levels of cytokines and chemokines to maintain homeostatic conditions, and exposure to NPs can influence their production.^[^ ^55^ ^]^ To evaluate the impact on secretion levels, we performed a multiplex assay using the ProcartaPlex Human Cytokine & Chemokine Panel 1A, 34‐plex, on both EoC and FT SoC models before exposure (baseline levels) and after 48 h of exposure to TiO_2_ NPs (33.33 µg cm^−2^). The heat map (**Figure** **7**A) provides an overview of cytokine and chemokine profiles before and after NP exposure. Under homeostatic conditions, the FT SoC model exhibited higher baseline levels of cytokines and chemokines compared to the EoC model. Notably, GRO‐α (*p* = 0.0009), GM‐CSF (*p* = 0.0325), and MCP‐1 (*p* < 0.0001) levels were significantly elevated in the FT SoC model (Figure 7B). These cytokines can modulate fibroblast metabolism and stimulate the synthesis of ECM components, which are essential for closely mimicking native skin architecture. The observed differences are attributed to the increased cellular complexity of the FT SoC model, including the presence of dermal fibroblasts, enhanced cell‐cell crosstalk, and continuous model proliferation and differentiation, which are absent in the epidermis‐only model. These findings are consistent with observations reported by other research groups.^[^ ^56^, ^57^ ^]^

Following NP exposure, the FT SoC model showed a more pronounced response compared to the EoC model. After stimulation with TiO_2_ NPs over 48 h, the production of GRO‐α (*p* = 0.0011) and MCP‐1 (*p* = 0.0008) was significantly downregulated compared to basal levels only in the FT SoC model. Studies show that TiO_2_ NPs can induce the release of pro‐inflammatory cytokines.^[^ ^58^, ^59^ ^]^ However, after 48 h of exposure, we do not observe significant signs of an inflammatory response. Interestingly, although not significant, we observe an increase in IL‐1Ra and IL‐23 levels in both models. IL‐1Ra is an anti‐inflammatory cytokine that competes with IL‐α and IL‐1β for receptor binding, contributing to inflammation resolution and playing a key role in skin homeostasis. Keratinocytes, and to a lesser extent, fibroblasts, are able to produce IL‐1Ra.^[^ ^60^ ^]^ Based on the effects observed on both models upon NP exposure, we conceive that NPs induce an early moderate acute inflammatory response that could trigger the production of endogenous IL‐1Ra to downregulate and resolve inflammation. Reijnders et al. observed a similar response using a FT human skin equivalent model following an environmental insult.^[^ ^61^ ^]^ Pro‐inflammatory mediators increased on the first day and subsequently returned to basal levels, showing early signs consistent with wound healing. Additionally, Klasson et al. reported a time‐dependent effect on cytokine and chemokine secretion following cobalt exposure in HaCaT cells, further emphasizing the transient nature of the inflammatory response to NPs.^[^ ^62^ ^]^

In a complementary methodology, Principal Component Analysis (PCA) was performed to uncover hidden information in the expression profiles of the cytokines and chemokines in EoC and FT SoC models before and after 48 h of exposure to TiO_2_ NPs. In the EoC model (Figure 7C), baseline and exposed samples clustered closely but remained distinguishable, with IL‐8, MCP‐1, and IL‐1Ra emerging as key contributors to the observed variance (Figure S5A, Supporting Information). The relatively subtle shift upon exposure suggests NPs slightly initiate an immunomodulatory effect in the epidermis‐only environment. In contrast, the FT SoC model (Figure 7D) shows a distinct clustering between exposed and unexposed samples to NPs, primarily associated with changes in MCP‐1, IL‐8, MIP‐1α, and GRO‐α (Figure S5B, Supporting Information). As previously mentioned, the significant production downregulation of GRO‐α in the FT SoC model after exposure to NPs makes GRO‐α the differentiator chemokine in the observed variance of both clusters. Overall, the distinguishable clustering underscores the complexity added by the dermal compartment. Leveraging the knowledge about inter‐sample variability and understanding which cytokines and chemokines drive the clustering, it is paramount to comprehend how each cluster interacts with the others. The cluster map (Figure 7E) shows three groups of cytokines and chemokines, suggesting coordinated changes in inflammatory mediators. Inside the clusters, it is noteworthy to stress out the proximity between (i) IL‐8 and MIP‐1*β;* and (ii) GRO‐α, GM‐CSF, and MCP‐1, which aligns with the observed expression profiles highlighted in Figure 7B. Although in a different cluster, a similar highlight can be done to the proximity between IL‐1Ra and IL‐23, which expressed a non‐significant increase in EoC and FT SoC models after exposure to NPs.

Altogether, this analysis emphasizes the complexity of the FT SoC model in recapitulating NP‐induced inflammatory responses, suggesting that it is a more physiologically relevant tool for evaluating the safety of NPs in skin‐related applications. In addition, the combined reduction in barrier integrity, reduced metabolic activity, and moderate inflammatory response observed upon TiO_2_ NP exposure illustrate early signs of tissue‐level stress and damage. These effects reflect not only superficial disruption but also deeper impairment of cellular function and paracrine communication. This underscores the importance of assessing multiple biological endpoints in complex, dynamic models like the EoC and FT SoC, and reinforces the potential of this platform as a valuable preclinical tool for predicting human skin responses to NP exposure.

### 2.7 Limitations and Comparative Effectiveness of Microfluidic SoC versus Traditional Static Models

Together, our results demonstrate that exposure to TiO_2_ NPs affects barrier integrity, cell viability, and skin permeability. This proof‐of‐concept study was designed to assess the initial performance of the EoC and FT SoC platforms using a single NP concentration and a 48 h exposure. To more comprehensively validate the predictive capacity of the EoC and FT SoC models, future studies should include dose‐response experiments (e.g., three or more concentrations), extended exposure durations (e.g., up to seven days), and mechanistic investigations such as assessment of reactive oxygen species (ROS) generation, DNA damage, and NP internalization. Additionally, further investigation is needed to elucidate the mechanisms underlying TiO_2_ NP cytotoxicity on both EoC and FT SoC models. This includes not only varying NP concentrations and exposure times, but also testing NPs with different chemical compositions to evaluate material‐specific effects. Moreover, the use of high‐resolution imaging techniques, such as transmission electron microscopy, could provide valuable insight into NP uptake and accumulation within skin layers, supporting a deeper mechanistic understanding of NP‐skin interactions. At the moment, studies are underway to include a human cell line (N/TERT‐2G) that cornifies to address the development of the stratum corneum.

It is important to stress that the developed microfluidic SoC model offers a substantial improvement in physiological relevance and predictive power compared to traditional static cell culture models for assessing NP safety. Traditional static models typically involve monolayer cultures or simple skin tissue constructs, which lack dynamic fluid flow and precise compartmentalization and often fail to fully replicate the structural and functional complexity of native skin. This can lead to limited insights into the dynamic cellular responses and biochemical gradients that are central to accurate toxicity and safety assessments.

In contrast, our model more closely mimics the skin microenvironment by integrating fluid flow, which maintains nutrient and oxygen gradients, removes cellular waste, and allows for the controlled introduction of NPs. This dynamic environment fosters continuous interaction between the epidermal and dermal compartments, better simulating how NPs might diffuse, interact with different skin layers, and ultimately influence cellular behavior. This flow‐driven system thus supports a more stable, sustained cellular response compared to static cultures, where conditions can become hypoxic or nutrient‐deprived, altering cellular responses and potentially skewing toxicity data.

The microfluidic system's comparative advantage is also evident in cytokine and chemokine profiling, as shown in the heat map analysis (Figure 7A). The FT SoC model revealed an intricate cytokine and chemokine response upon NP exposure that more accurately reflects the human skin's response to external stressors, which is critical for developing safer nanomaterials and for regulatory assessments. Traditional static models often fail to capture such a nuanced response due to the lack of tissue complexity and inter‐compartmental communication.

Beyond these advantages over static models, the device also introduces innovations that distinguish it from previously published SoC systems. Our modular design allows straightforward implementation of both epidermis‐only and FT skin configurations within a single platform, facilitating comparative studies and screening applications. The integration of a removable TEER system enables longitudinal, non‐invasive monitoring of barrier integrity during dynamic perfusion, providing real‐time assessment capability. Moreover, fabrication using PMMA overcomes key limitations associated with PDMS, including the absorption of small molecules and high gas permeability, while supporting scalable and reproducible manufacturing.^[^ ^63^, ^64^ ^]^ The device also incorporates multiplex cytokine and chemokine profiling to assess inflammatory responses, expanding its utility for nanotoxicological assessment.

The inclusion of immune system cells (e.g., Langerhans cells and dermal dendritic cells) in the model can also enhance its physiological relevance and is essential for gaining deeper insight into the immunomodulatory profile of NPs and their mechanisms of action, as many of the cytokines and chemokines secreted are involved in the recruitment of immune cells.

In summary, the microfluidic SoC model surpasses traditional static models by offering a more realistic and predictive representation of human skin physiology. The model's compartmentalized environment, layered structure, and dynamic flow give significant benefits in capturing the intricate interactions and reactions to NPs, resulting in a higher level of fidelity in NP safety evaluation. The microfluidic model is positioned as a superior platform for toxicity screening and as a crucial tool in the safety evaluation of future nanotechnologies due to its increased physiological relevance and predictive capacity.

## 3 Conclusion

The main aim achieved with this work was the development and optimization of an innovative microfluidic system that recapitulates the in vivo epidermis and FT skin growth for NP safety assessment.

Fluid dynamics simulations reveal that in the constructed model, the shear stress induced is 0.06 dyne cm^−2^, a value that is known not to affect keratinocyte growth. Medium degasification and bubble traps were employed in the system since air bubbles were impairing tissue formation. The reproducibility and integrity of the model were supported by the use of well‐characterized HaCaT keratinocyte and Hs27 fibroblast cell lines, regularly tested for contamination and employed within early passage numbers.

Results collectively indicate that seeding keratinocyte and fibroblast cells at a density of 2 × 10^6^ and 5 × 10^6^ cells cm^−2^, respectively, using a collagen type I concentration of 6.0 mg cm^−3^, employing cellulose‐based O‐rings on devices with a PET porous membrane of 1.0 µm pore size, represent the optimal conditions for establishing a reliable FT SoC model. This configuration mimics the native environment of human skin, allowing the investigation of the effect of TiO_2_ NP exposure on cellular viability, permeability, and barrier integrity.

TEER measurements, metabolic activity, paracellular permeability, and histological and immunohistochemical analyses confirmed the formation of a distinct dermal compartment and a stratified epidermis, indicating successful tissue growth.

Exposure of TiO_2_ NPs to both the EoC and FT SoC models reveals a reduction in epidermal barrier integrity, a decrease in metabolic activity, and an increase in paracellular permeability, highlighting the potential cytotoxic and barrier‐disruptive effects of these NPs. Overall, the FT SoC exhibited higher basal secretion levels of cytokines and chemokines, consistent with the increased complexity and proliferative state of the model, helping to more closely mimic the native structure of human skin. After 48 h of NP exposure, we observed an upregulation of the anti‐inflammatory cytokines IL‐1RA and IL‐23, which contribute to the resolution of inflammation and mediate tissue repair following damage. PCA analysis and hierarchical correlation of cytokines and chemokines in EoC and FT SoC highlight the additional complexity of the dermal compartment in the FT SoC.

In conclusion, this microfluidic device stands out as a relevant model for conducting comprehensive safety assessment studies of NPs. Its ability to mimic the complex architecture and function of in vivo skin makes it a useful tool for understanding the interactions and potential risks associated with NP exposure, thereby contributing significantly to the field of nanotoxicology and ensuring the development of safer nanomaterials.

Nonetheless, a key limitation of the current SoC model is the use of the HaCaT keratinocyte cells, which are known to lack full cornification and do not form a functional stratum corneum. While these cells provided a stable and reproducible platform for initial model development and toxicological testing, future work is already underway to incorporate more physiologically representative human skin models, such as cornifying keratinocyte cell lines like N/TERT‐2G, to enhance the structural and functional fidelity of the system.

## 4 Experimental Section

### Chemicals and Materials

All chemicals and materials used in this study were obtained from commercial suppliers, stored, and handled in accordance with the manufacturer's instructions.

Microfluidic chips were custom‐fabricated using biocompatible PMMA sheets with different thicknesses (ME30‐SH‐, GoodFellow), PET porous membrane (2000M12/811N401, it4ip), and double‐sided tape (12SQ‐12‐467MP, 3 m). Inlets and outlets were connected via polytetrafluorethylene (PTFE) tubing (008T16‐050‐20, Antylia Scientific) and Tygon tubing (95702‐00, Antylia Scientific). Syringes (SS+20L1, Terumo) were used for medium perfusion, with flow controlled by a syringe pump (NE‐1200, NORLEQ) operating at a flow rate of 1 µL min^−1^. Nalgene vacuum chamber (5305‐0609, Thermo Scientific) and bubble traps (LVF‐KBT‐S, Darwin microfluidics) were used for gas management.

Cell culture media and supplements included DMEM High‐glucose (11 965 092, Gibco), Ham's‐F12 Nutrient Mixture (21 700 075, Gibco), FBS (A4736401, Gibco), penicillin/streptomycin solution (15 140 122, Gibco), sodium pyruvate (11 360 070, Gibco), HEPES buffer (15 630 080, Gibco), and L‐glutamine (A2916801, Gibco). For epidermal differentiation, additional supplements were used: cholera toxin (C8052, Merck), apo‐transferrin (T1147, Merck), hydrocortisone (H0888, Merck), EGF (SRP3027, Merck), TGF‐β (PHG9211, Gibco), and insulin (I9278, Merck).

Cellular matrix components included collagen type I from bovine skin (C2124, Merck) and collagen type IV from human placenta (C7521, Merck).

Viability, metabolic activity, and permeability assessments were conducted using the Live/Dead cell imaging kit (R37601, Invitrogen), PrestoBlue High Sensitivity Cell Viability Reagent (P50201, Invitrogen), and Lucifer Yellow CH Di lithium salt (L453, Invitrogen), respectively. Paraformaldehyde (4%, J61899.AP, Thermo Scientific Chemicals) and 10% neutral buffered formalin (HT501128, Merck) were used for tissue fixation. Phosphate‐buffered saline (PBS, 10 010 023, Gibco) was used for sample washing and dilution procedures. For immunohistochemical analyses, specific primary antibodies were used: anti‐cytokeratin 14 (MA511599, Invitrogen), anti‐cytokeratin 10 (MA106319, Invitrogen), and anti‐loricrin (PA530583, Invitrogen). Cytokine and chemokine quantification were carried out using the ProcartaPlex Human Cytokine & Chemokine Panel 1A, 34‐plex kit (EPX340‐12167‐901, Invitrogen).

TiO2 NPs (7920DL, SS Nano) were used for exposure assays. For dispersion studies, Milli‐Q water and BSA (A9418, Merck) were employed.

### Cell Culture

Spontaneously transformed human keratinocytes HaCaT cell line (300 493, CLS Cell Lines Service GmbH; RRID: CVCL_0038) and human foreskin fibroblasts Hs27 cell line (CRL‐1634, ATCC; RRID: CVCL_0335) were used as human epidermis and dermis models, respectively. The cell lines were thawed and maintained in complete culture medium–DMEM High‐glucose supplemented with 10% FBS and 1% penicillin/streptomycin solution. Additionally, the Hs27 cell line culture medium was also supplemented with 1% sodium pyruvate. The cells were routinely subcultured in culture flasks when they reached a confluence of 80–90%. Furthermore, both cell lines were routinely tested for mycoplasma contamination using a PCR‐based assay and were found to be negative.

### Cell Culture: Characterization of HaCaT and Hs27 Cell Lines

The HaCaT and Hs27 cell lines were used and characterized between passages 3 and 14, with passage one corresponding to the first culture performed in the laboratory. The HaCaT keratinocyte cells were obtained at passage 32, and the Hs27 fibroblast cells at passage 7. Cell morphology and confluence were evaluated using phase contrast microscopy, and cell viability was assessed with a Live/Dead cell imaging kit under a fluorescence microscope at 24, 48, and 72 h post‐seeding. For these assays, ≈20 000 cells were seeded per well in 96‐well plates, corresponding to a density of 60 × 10^3^ cells cm^−2^ (based on an average well surface area of 0.33 cm^2^). The images obtained from these analyses were processed using Image J and used to ensure the reliability of the cell lines for further experiments and to confirm their suitability as models for epidermal and dermal studies.

### Device Design and Fabrication

The microfluidic device was designed using CorelDRAW Graphics Suite X8 software and fabricated by assembling precision‐cut PMMA sheets and a porous PET membrane. The fabrication process was performed as follows: three PMMA sheets with thicknesses of 8.0 mm (upper layer), 1.0 mm (middle layer), and 0.5 mm (lower layer) were selected, and a PET membrane with a pore size of 1.0 µm was used. The upper PMMA sheet (8.0 mm) was laser‐cut to define two circular chambers: a central cell culture chamber with a diameter of 6.5 mm and a smaller adjacent chamber for TEER measurements with a diameter of 5.0 mm. The middle PMMA sheet (1.0 mm) was patterned to create a straight microfluidic channel measuring 31.0 mm in length, 1.0 mm in width, and 1.0 mm in height. The PET membrane was carefully aligned and sandwiched between the patterned upper and middle PMMA sheets, serving as a permeable barrier between the cell culture chamber and the microfluidic channel. The lower PMMA sheet (0.5 mm) was then bonded beneath the middle layer to fully seal the channel structure. Double‐sided adhesive tape was used to ensure precise alignment and tight bonding between all layers. Inlet and outlet ports (2.0 mm diameter) were incorporated into the upper layer. The inlets were connected to the culture medium via PTFE tubing attached to bubble traps, which were linked to syringes filled with medium using Tygon tubing. The outlets were connected to reservoir flasks to collect the perfusion medium. Continuous perfusion of the cell culture medium was maintained throughout the experiments using a syringe pump at a controlled flow rate of 1.0 µL min^−1^. The entire setup was kept inside a standard humidified incubator to ensure a stable physiological temperature and CO_2_ levels, thereby creating a stable culture environment. Additionally, a detachable PMMA support system was designed and fabricated to hold up to six devices simultaneously. This support allows convenient handling during culture and facilitates direct microscopic inspection of the devices without disrupting the experimental conditions.

### Device Design and Fabrication: Modeling shear Stress Dynamics and Transepithelial Electrical Resistance Performance

A finite element modeling study was done to evaluate the fluidic and electrical characteristics of the designed device by COMSOL Multiphysics software version 5.4 (COMSOL AB, Sweden). In computational simulations, models often rely on assumptions or approximations about details that are not fully known. For instance, the surface of the tissue may be assumed to be smooth, flat, and non‐slip. In reality, there is always some roughness, and shear stress can be higher in some spots and lower in others. However, the average shear stress rate is correct, and knowing the velocity of the flow in the chamber, the profile at the bottom of the chamber could be computed to understand how it can affect cell growth and differentiation. Accordingly, one study estimated the shear stress on the cells caused by the perfusion flow (1 µL min^−1^ flow rate and 1 mPa s^−1^ viscosity), and another one evaluated the TEER performance with a configuration of four electrodes (medium with a conductivity of 1.5 S/m, TEER resistance in the range of 25.0 to 500.0 Ohms cm^2^, and excitation voltage of 1.0 V applied between two vertical 0.5 mm diameter electrodes of neighboring chambers, where current sourcing electrode pair was the inner one and voltage monitoring pair the outer one). Hydrodynamic studies were based on laminar flow physics, while TEER used direct electrical currents.

### Reconstructed Human Epidermis and Full‐Thickness Skin‐on‐Chip: Device Preparation

First, the fabricated devices were wiped with 70% ethanol and dried in the oven for 30 min at 65 °C, followed by UV radiation for another 30 min to sterilize them. Once sterilized, the devices were ready for cell culture to reconstruct the tissues.

### Reconstructed Human Epidermis and Full‐Thickness Skin‐on‐Chip: Epidermis‐On‐Chip

The chambers were coated with a collagen type IV solution (60 µL at 0.5 mg mL^−1^ in serum‐free DMEM). After 1 h 30, the chambers were washed with the complete culture medium, and HaCaT keratinocyte cells were seeded at a density of 5 × 10^6^ cells cm^−2^ with medium flow completely stopped to allow cell attachment onto the membrane.

Thus, 2 h post‐seeding, the syringe pump drove a steady flow of the degassed differentiation culture medium, and microfluidic cultures were maintained at standard cell culture conditions (37 °C, 5% CO_2_). The differentiation culture medium consists of DMEM and Ham's‐F12 Nutrient Mixture in a 3:1 ratio, supplemented with 2 ng mL^−1^ cholera toxin, 5 µg mL^−1^ apo‐transferrin, 0.4 µg mL^−1^ hydrocortisone, 1 ng mL^−1^ EGF, 2 ng mL^−1^ TGF‐β, 5 µg mL^−1^ insulin, and 5% of fibroblast‐conditioned medium.

To establish an ALI on the apical side of the HaCaT cultures, after 48 h, the culture medium was carefully aspirated from the chambers while the perfusion was maintained constantly for a keratinocyte growth period of 12 days. Additionally, low incubator humidity was sustained in the last 3 days of culture to promote tissue differentiation and enhance subcutaneous hydration and permeability.^[^ ^29^ ^]^

As a biological control, static epidermis reconstructions were performed in 24‐well cell culture inserts with a PET membrane of 1.0 µm porosity (9321012, CellQART). Instead of maintaining a continuous flow, the basal medium was replaced every two days to ensure a fresh supply of nutrients. Detailed results for the static conditions were obtained by replicating the culture conditions described above, with the same differentiation culture medium and incubation times.

### Reconstructed Human Epidermis and Full‐Thickness Skin‐on‐Chip: Full‐Thickness Skin‐On‐Chip

The human FT skin was reconstructed using an acellular collagen type I‐based dermal matrix. The first step was preparing a pre‐mix solution composed of serum‐free DMEM, L‐Glutamine, penicillin/streptomycin, HEPES buffer, and FBS. Then, a 6.0 mg cm^−3^ collagen solution from bovine skin was mixed with the complete culture medium and the pre‐mix solution to make the acellular dermal matrix to add to each well. Both preparations were performed in a box filled with ice. After 1 h 30 of incubation, cellulose‐based O‐rings were placed on the top of the acellular dermal matrix to prevent collagen contraction.

For the dermal reconstruction, Hs27 fibroblast cells were seeded at a density of 2 × 10^6^ cells cm^−2^ per chip. Accordingly, the cellular layer was prepared by combining the collagen type I solution, the previously prepared pre‐mix solution, and the Hs27 cell suspension, which was then added to each well. After 2 h, a continuous flow of complete DMEM was initiated, and the microfluidic system was maintained at 37 °C in a humidified incubator with 5% CO_2_. Finally, 48 h later, the epidermal layer was recreated following the steps previously described in Section Reconstructed Human Epidermis and Full‐Thickness Skin‐on‐Chip:Epidermis‐On‐Chip.

For static controls, the approach was similar to that used for epidermis reconstruction but applied to the FT skin model. Static conditions were established using 24‐well cell culture inserts with a PET membrane of 1.0 µm porosity. In this setup, the basal medium was replaced every two days. This allowed for comparison with the microfluidic system while replicating the culture conditions described for the FT skin reconstruction.

### Human Epidermis and Full‐Thickness Skin Characterization: Transepithelial Electrical Resistance Measurements

The electrical resistance of the EoC and FT SoC and respective static controls was measured every 2 days from day 0 at ALI using the in‐house four‐electrodes configuration developed, connected to an Epithelial Volt Ohm Meter (EVOM2, World Precision Instruments) to assess the epidermal barrier integrity. The contribution of the membrane and culture medium is accounted for by subtracting the electrical resistance of the membranes without cells from the sample values and normalizing it to the culture surface area by multiplying the net electrical resistance by the total surface area covered by the keratinocyte barrier to calculate the TEER in Ohms cm^2^. For this, one fluidic chamber was kept free of cells during the experiments as a control, and the TEER value was measured in parallel and subtracted from that of the TEER values of the chambers with cells. Furthermore, the length of the electrode tips is unequal, allowing the longer tips to enter the smaller chamber, accessing the microfluidic channel, and the shorter tips to enter the chambers where the tissue grows. It should be noted that TEER is not a directly measured quantity but a calculated parameter derived from the resistance values recorded by the EVOM2. The FEM confirmed that the intrinsic resistance contribution of the microfluidic chip (1.5 kOhms) was effectively captured and corrected for through this blank subtraction procedure, ensuring that the resulting TEER values are directly comparable to those obtained from standard measurements.

### Human Epidermis and Full‐Thickness Skin Characterization: Metabolic Activity

After epidermal differentiation time in ALI, metabolic activity was assessed using a PrestoBlue solution (1:10 in cell complete culture medium) and incubated at 37 °C and 5% CO_2_, protected from direct light. Five incubation times were tested to optimize sensitivity, with readings taken to ensure the fluorescence signal was within the linear range. The chosen time point for final readings was 4 h. The fluorescence was measured using a BioTek Synergy H1 microplate reader, with an excitation wavelength of 560 nm and an emission wavelength of 590 nm.

### Human Epidermis and Full‐Thickness Skin Characterization: Paracellular Permeability

The apical‐to‐basolateral transport flux, which is correlated to the paracellular transport route, has been quantified by measuring the permeability of the skin to Lucifer Yellow as a flux tracer. To measure this, the Lucifer Yellow solution was added to the top of the differentiated tissues, and the paracellular flux of the tracer molecule was quantified by serially sampling fluid from the microfluidic channels and basolateral compartment in inserts at different time points. The Lucifer Yellow concentration in the collected samplings was determined using the BioTek Synergy H1 microplate reader, with excitation/emission wavelengths set at 428 nm and 540 nm, respectively. Simultaneously, TEER measurements were taken to monitor the skin's barrier integrity.

### Human Epidermis and Full‐Thickness Skin Characterization: Histological and Immunohistochemical Analyses

To analyze the morphology of the epidermis and FT skin structure, the tissues were subjected to HE staining. First, the skin tissue samples were fixed in 10% neutral buffered formalin and separated from the microfluidic chips and inserts by cutting along the skin's edge with a scalpel. Then, the samples were washed with PBS and embedded in paraffin. After sectioning on glass slides with a thickness of 5.0 µm, they were deparaffinized in xylene, rehydrated in decreasing ethanol concentrations, and finally stained with HE.

In turn, immunohistochemistry was performed to examine the growth and differentiation of the reconstructed epidermis layer by the distribution of K14 (basal cell layer), K10 (early differentiation), and LOR (late differentiation). Primarily, the skin tissue samples were fixed in 4% paraformaldehyde for 3 h at room temperature. Next, the samples were washed with PBS and processed for paraffin embedding. After sectioning the samples on glass slides with a thickness of 5.0 µm, they were deparaffinized, subjected to heat‐induced epitope retrieval, and blocked with 3% hydrogen peroxide (H_2_O_2_) to inhibit endogenous peroxidase activity. The slides were then incubated with primary antibodies diluted as follows: 1:1600 anti‐cytokeratin 14 monoclonal antibody, 1:200 anti‐cytokeratin 10 monoclonal antibody, and 1:500 anti‐loricrin polyclonal antibody. After washing, the slides were incubated with appropriate secondary antibodies conjugated with horseradish peroxidase (HRP) specific to the primary antibodies, and the peroxidase activity was visualized using a 3,3′‐diaminobenzidine (DAB) substrate kit, resulting in a brown color at the sites of antigen‐antibody binding. Finally, the slides were counterstained with hematoxylin to visualize cell nuclei.

### Titanium Dioxide Nanoparticles: Physicochemical Characterization and Dispersion Optimization of Titanium Dioxide Nanoparticles

TiO_2_ NPs were characterized using several advanced techniques. This fine white powder comprises titanium oxide particles with a D50 ranging from 10 to 30 nm and a surface area of ≈50.0 m^2^ g^−1^.

XRD patterns were recorded with an XPert Pro PANalytical diffractometer using Cu K‐α radiation (1.54184 Å, 45.0 kV, 20 mA) to determine the crystalline structure. A structural analysis was also performed by WITec's Raman spectrophotometer in backscattering geometry, employing a 50x objective and a 532.0 nm ion argon laser at 0.5 mW power, using a 600.0 g mm^−1^ grating. For surface analysis, XPS was performed using a Thermo Scientific ESCALAB 250Xi system. Survey spectra were collected at 100.0 eV pass energy, while individual element spectra were obtained at 40.0 eV with a 0.1 eV energy step. The sample was cleaned with an argon cluster ion gun (4.0 keV, cluster size 1000 atoms) and mounted on a sample holder with double‐sided carbon tape. XPS data were processed using Avantage software, applying Shirley‐type background subtraction and quantification based on sensitivity factors from the Avantage library. Lastly, the particle size and morphology of the dry powder and TiO_2_ NPs suspension in ultra‐pure water (without dispersion) were examined using a JEOL 2100, 200.0 kV, scanning transmission electron microscope.

The aim of the dispersion of the TiO_2_ NPs is the complete deagglomeration of the particles in the culture medium used in SoC to achieve nanometric particle sizes comparable to those found in sunscreens. The strategy employed for the dispersion had two basic procedures: powder dispersion by sonication in Milli‐Q water and dilution of the stock suspension in the complete culture medium using BSA as a stabilizing agent. Mean particle size and PDI were assessed by DLS using a HORIBA Scientific ZetaSizer Nano ZS‐100 at 25 °C using standard 10.0 mm disposable optical polystyrene cuvettes. DLS measurements were conducted in triplicate for each condition, with the number and duration of sub‐measurements automatically determined by the instrument's software. Before establishing the sonication protocol, a calorimetric method was used to calculate the specific energy as well as the sonication power transferred to the suspension.

For the powder dispersion by sonication, the TiO_2_ NPs were diluted in Milli‐Q water to obtain different concentration suspensions (0.2, 0.5, 1.0, and 2.0 mg mL^−1^). Using Branson Ultrasonic Disintegrator Mod. 450, the tip (Ti 13.0 mm) was immersed 2.0 cm into the suspensions, dispersing the NPs by ultrasound at 25 °C for different times (15 and 30 min) in pulsed mode (8 seconds on/2 seconds off) across varying specific energies (1.15 × 10^4^, 1.89 × 10^4^, and 2.91 × 10^4^ KJ m^−3^). After optimizing the dispersion conditions in Milli‐Q water (0.5 mg mL^−1^, 1.15 × 10^4^ KJ m^−3^, and 30 min of pulsed mode), the 24 h stabilized TiO_2_ NPs stock solution was diluted to a concentration of 100.0 µg mL^−1^ in the complete DMEM, and DLS also determined the size distribution. BSA at a final concentration of 0.6 mg mL^−1^ in PBS was previously added to the suspension. In addition, the particle size and morphology of the suspension in ultra‐pure water (dispersion condition) and in the complete culture medium were analysed by STEM.

### Titanium Dioxide Nanoparticles: Exposure of Titanium Dioxide Nanoparticles on Epidermis and Full‐Thickness Skin‐On‐Chip

The EoC and FT SoC and respective static controls established on day 12 at ALI were incubated for 48 h with 100 µL of the TiO_2_ NPs stock solution diluted to a concentration of 33.33 µg cm^−2^ in complete DMEM. This concentration and time point were chosen based on previously reported experience, as they reflect typical levels of active ingredients found in commercial sunscreens, allowing to evaluate the biological effects of the TiO_2_ NPs in a context similar to their potential exposure.^[^ ^49^ ^]^

TEER measurements were made every 24 h to assess the integrity of the epidermal barrier over time. Histological analyses, PrestoBlue, and Lucifer Yellow assays were performed to evaluate the impact of exposure to the TiO_2_ NPs on the skin morphology, metabolic activity, and permeability, respectively.

### Cytokines and Chemokines Quantification

The medium that traversed the different microfluidic channels of the chips during the EoC and FT SoC assays, both before and after 48 h of exposure to TiO_2_ NPs, was collected and frozen for subsequent analysis and quantification of cytokines and chemokines.

The ProcartaPlex Human Cytokine & Chemokine Panel 1A, 34‐plex kit was used according to the manufacturer's specifications. Belysa Immunoassay Curve Fitting Software V1.2.2 (Software Solutions, Millipore) was used to analyze and fit data from immunoassay experiments.

A data‐driven workflow was implemented to extract biological insights and map relationships within cytokine and chemokine data.^[^ ^65^ ^]^ The workflow was developed using Python (V3.9.12) in a Jupyter Notebook environment (V6.5.4), using the following libraries: NumPy (V1.23.5) and Pandas (V1.5.3) for data manipulation; Matplotlib (V3.6.3) and Seaborn (V0.12.2) for data visualization; SciPy (V1.10.1) for hierarchical clustering and dendrogram generation; and Scikit‐learn (V1.2.0) for feature scaling and PCA. Two datasets were prepared using Luminex technology: median fluorescence intensity (MFI) raw values and the curve‐fitted analyte concentrations. Each original dataset contains 36 observations on 34 protein targets, with measurements spanning a wide range of biomarkers for EoC and FT SoC models before and after 48 h of exposure to TiO_2_ NPs. Each dataset comprises experimental labels regarding the (i) used SoC model (i.e., EoC versus FT SoC) and (ii) the exposure to NPs (i.e., with and without TiO_2_ NP exposure), streamlining the exploration of clusters within samples and cytokines/chemokines. The MFI‐based dataset served as the primary input for sample clustering analysis. PCA was applied to visualize inter‐sample variance across each independent SoC model (i.e., EoC and FT SoC models were treated separately). Key biomarkers contributors to the first and second principal components (PC1 and PC2) were identified, highlighting the cytokines and chemokines responsible for driving inter‐sample variation and enabling the detection of biologically meaningful clusters. The curve‐fitted analyte concentrations database served as the primary input for cytokines and chemokines clustering (i.e., for identifying biomarkers exhibiting similar expression profiles). After pre‐processing and normalizing the data, hierarchical clustering was performed using a correlation‐based distance metric and the weighted linkage method. Then, a heatmap integrating the mentioned techniques, also defined as a cluster map, was generated. The developed code and datasets are provided in Supplementary Information.

### Statistical Analysis

Statistical analyses were performed using the GraphPad Prism 10.2.1 software program. Data for TEER, metabolic activity, and apparent permeability were tested for normality using the D'Agostino‐Pearson test and found to follow a normal distribution. Independent unpaired two‐tailed t‐tests were used for each pairwise comparison: EoC versus static epidermis, FT SoC versus static FT skin, EoC control versus EoC exposed to TiO_2_, and FT SoC versus FT SoC exposed to TiO_2_. Although some control groups were reused across different comparisons (i.e., EoC control and FT SoC control), each test was performed independently and visualized in separate figures. Cytokine results were subjected to one‐way ANOVA analyses. Where significant differences were found, the Tukey´s multiple comparisons post hoc test was performed. Results were considered statistically significant at a p‐value of < 0.05. Measurements are reported as means ± SD and significant *p* values: *p < 0.05, **p < 0.01, and ****p < 0.0001. Statistical plots and graphs were created to summarize and visualize the data.

## Conflict of Interest

The authors declare no conflict of interest.

## Author Contributions

S.C. and A.C. performed methodology, investigation, visualization, validation, formal analysis, wrote original draft, reviewed, and edited the manuscript; F.L. performed methodology, investigation, wrote, reviewed, and edited the manuscript; J.M. performed methodology, software, wrote, reviewed, and edited the manuscript; A.A. performed methodology, investigation, wrote, reviewed, and edited the manuscript; C.M. wrote, reviewed, and edited the manuscript; E.A.‐M. wrote, reviewed, and edited the manuscript, and performed funding acquisition; A.R. performed conceptualization, wrote, reviewed, and edited the manuscript, and performed supervision.

## Supporting information



Supporting Information

Supporting Information

## Data Availability

The data that support the findings of this study are available from the corresponding author upon reasonable request.
