# Supplementary material for: A Novel Microfluidic System for 3D Epidermis and Full‐Thickness Skin Growth for Nanoparticle Safety Assessment
Source: Adv Healthc Mater. 2025 Nov 2;15(6):e02518. doi: 10.1002/adhm.202502518 (PMC12892020; doi:10.1002/adhm.202502518)
Supplement: Supplementary file 1 — Supporting Information [file ADHM-15-0-s002.docx]

Supporting Information

A Novel Microfluidic System for 3D Epidermis and Full-Thickness Skin Growth for Nanoparticle Safety Assessment

Samantha Costa, Ana B. Carneiro, Filipa Lebre, João Meneses, Alar Ainla, Cacilda Moura, Ernesto Alfaro-Moreno, and Ana R. Ribeiro*

Experimental validation of the microfluidic shear stress by Particle Image Velocimetry (PIV):

Particle Image Velocimetry (PIV) measurements were performed to validate the computational fluid simulation results experimentally. The flow experiments used 20 µm polyamide tracer particles suspended in water. Before use, the particles were sonicated in water for 15 min to ensure uniform dispersion. The suspension was driven through the microchannel of the chip at a flow rate of 1.0 µL/min. A total of 100 sequential bright-field images were acquired at 4x magnification, with a calibration factor of 5.0849 px/µm, and a time step of 12 s between frames. The experiments were conducted at room temperature, corresponding to a dynamic viscosity of μ = 0.00089 Pa s for water. PIV analysis was performed in MATLAB V24.2 (R2024b) on PCWIN64 using the PIVlab toolbox V3.9. The software was used with its standard settings for velocity field computation and shear rate extraction. The region of interest (ROI) was defined at the junction between the microchannel and the chamber, where the tracer particles flowed from the channel into the chamber, ensuring consistent particle tracking in an area of interest for the flow characterization. PIVlab was set to export the shear rate values computed from the velocity field gradients, which correspond to the magnitude of the 2D shear rate tensor as calculated internally by PIVlab. For each frame, the shear rate values within the ROI were exported to Excel files (.xls). A custom MATLAB script concatenated all shear rate data from the 100 frames and converted them to shear stress values using Equation 1:

τ = μ × γ̇

where μ is the dynamic viscosity of water at 25 °C, and γ̇ is the local shear rate from PIVlab (in s⁻¹). The maximum shear stress value across all frames was then identified and reported.

The maximum shear stress obtained from the experimental data was 155 µPa, which is in close agreement with the fluid flow simulation prediction of approximately 150 µPa.


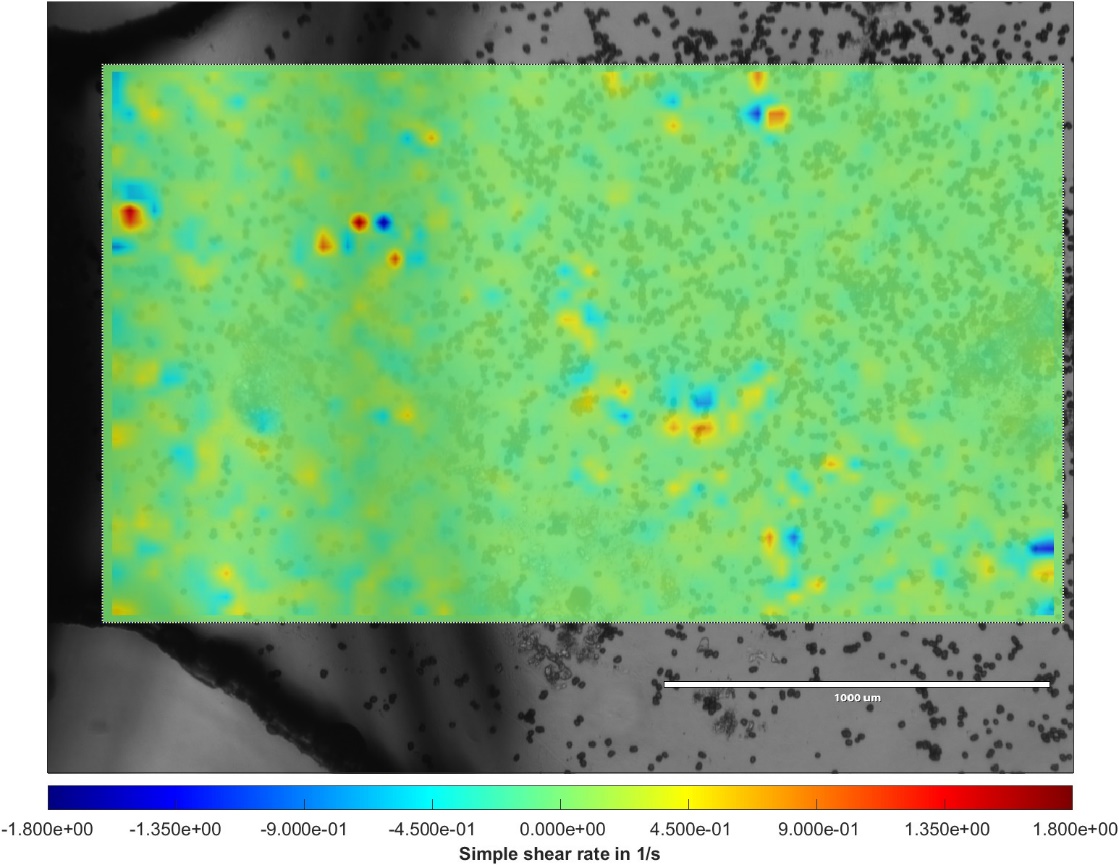


Figure S1. Particle Image Velocimetry shear rate in the microfluidic device. The color map represents the local shear rate within the ROI at the junction between the microchannel and the chamber, with a scale bar of 1000 µm. The data correspond to one of the 100 sequential frames used for shear stress calculation.

Electrical simulation of TEER measurements:

ln order to evaluate experimentally measured electrical resistance dependence on the TEER value of the cell layer, electrical simulation of the chip and electrodes were performed using Finite Element Modelling (FEM) with software package COMSOL Multiphysics. More specifically, the model was based on the physics of electrical currents in 3D geometry and was stationary (time-independent). Geometry represented channels and chambers with homogeneous electrical conductivity of 1.5 S/m, corresponding to the cell culture medium. The tissue was modeled as a thin layer with uniform TEER resistance. Four electrodes were placed into the chambers corresponding to the experimental setup. Following boundary conditions were used: i) all walls of the channels and chambers were insulating, ii) walls of the electrodes were at constant potential, iii) voltage sensing electrode had free floating potential and iv) potential between current sourcing electrodes was set to 1 V (this is arbitrary value, since it is linear system, where currents are always directly proportional to the voltage). After the simulation, the current through the sourcing electrodes and the potential between the sensing electrodes were determined. The ratio between the potential difference of the sensing electrodes and the current of the sourcing electrodes corresponds to the resistance as measured experimentally by EVOM. By varying the TEER parameter of the cell layer, a relationship was established between the measured resistance value and TEER. Additionally, we determined the variation in current density through the homogeneous cell layer. If TEER value is higher, measured resistance is more dominated by the TEER, the current density variation over the cell layer is smaller meaning that different areas of the cell layer contribute more uniformly to the average measured resistance value, in contrast to low TEER value, when regions with higher current density would have higher influence to the measured resistance.


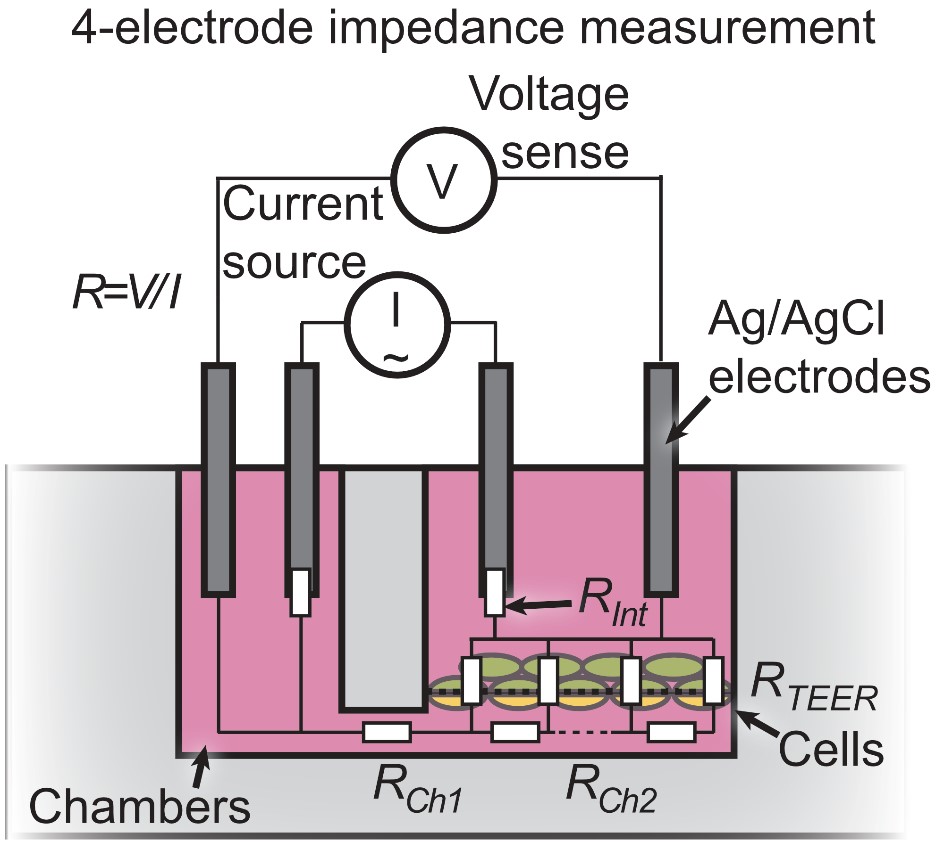


Figure S2. Conceptual illustration of the on-chip TEER measurement. A four-point measurement configuration with Ag/AgCl electrodes was used, where two electrodes drive an AC current (in the case of EVOM2, 10 µA at 12.5 Hz) and two other electrodes sense the voltage. This configuration eliminates the effect of the complex, non-linear interfacial impedance of the electrodes (R_Int_), allowing for a more accurate measurement of the fluidic medium and cells with a simpler linear impedance. Microfluidic channels with small dimensions can have significant own resistance (R_Ch1_ and R_Ch2_), and they can also influence the current distribution through the cell layer (R_TEER_), which is usually not a concern in well plates where R_Ch2_ is low compared to the cells.

Human Fibroblasts and Keratinocytes Characterization:


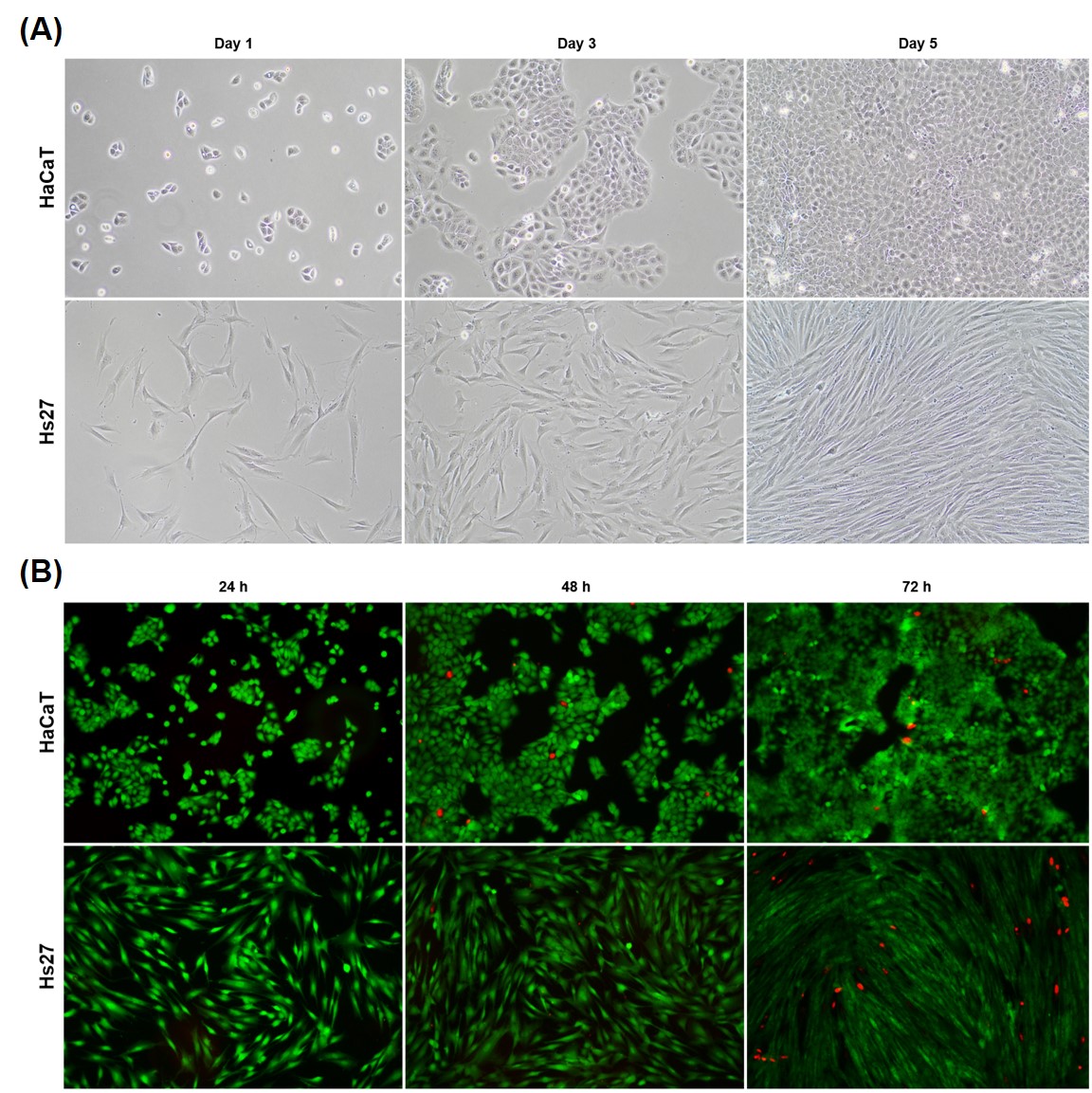


Figure S3. Characterization of HaCaT and Hs27 cell lines: (A) Phase contrast microscopy images showing the proliferation of HaCaT keratinocyte and Hs27 fibroblast cells on days 1, 3, and 5 of subculturing. 200x magnification. (B) Live/Dead assay results for both cell lines at 24, 48, and 72 h post-seeding, with green indicating live cells and red indicating dead cells. 200x magnification.

**TEER measurements setup**:

TEER measurement was performed with EVOM2 reader using a four-electrode configuration. For consistent and reproducible electrode placement, a custom holder specific to the chips was designed for the measurements. The electrode holder was transferable between chips to perform measurements at the required time points. To ensure that electrodes were always positioned at the same location in the chambers, alignment features were incorporated into the holder. Two electrodes were placed near the center of each chamber. The Holder was designed in free, open-source software OpenSCAD and 3D printed using a stereolithographic (SLA) printer, Form 3 (Form Labs), with Grey Pro V4 resin. Thereafter, silver wires (0.5 mm diameter, 3.5 mm length, Merck) were attached using epoxy glue, and interfacing electrical wires were soldered to the external ends of the silver wires. To form Ag/AgCl electrodes, silver wires were immersed in 0.1 M HCl, and using a platinum counter electrode (Merck) as a cathode, a constant current of 1.1 mA (current density 10 mA cm^-2^) was applied for 1 minute by a source meter (Keithley).


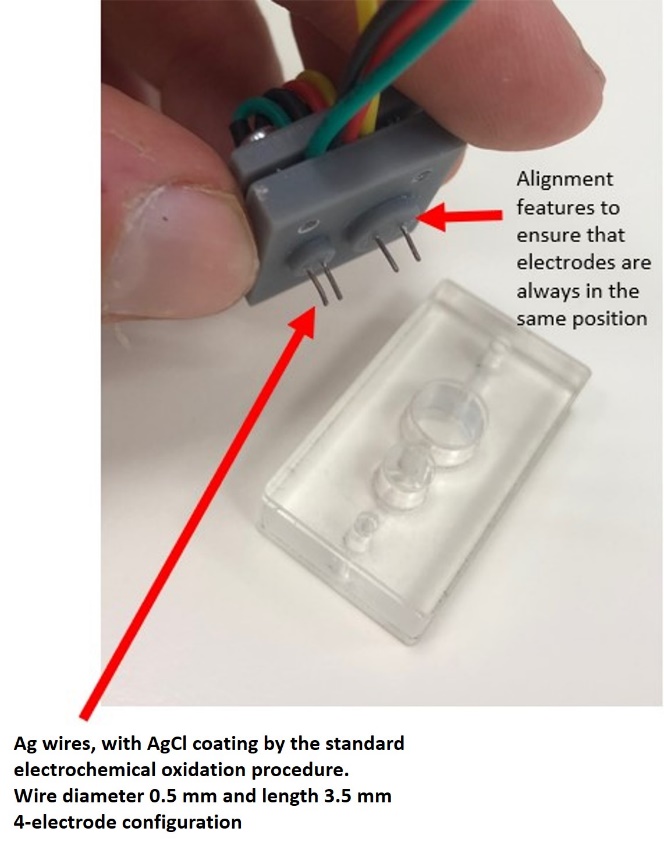


**Figure S4.** Reusable electrode holder for TEER measurements in EoC and FT SoC. The electrode holder is 3D printed and equipped with four Ag/AgCl electrodes.

**Cytokines and Chemokines Analysis and Quantification:**


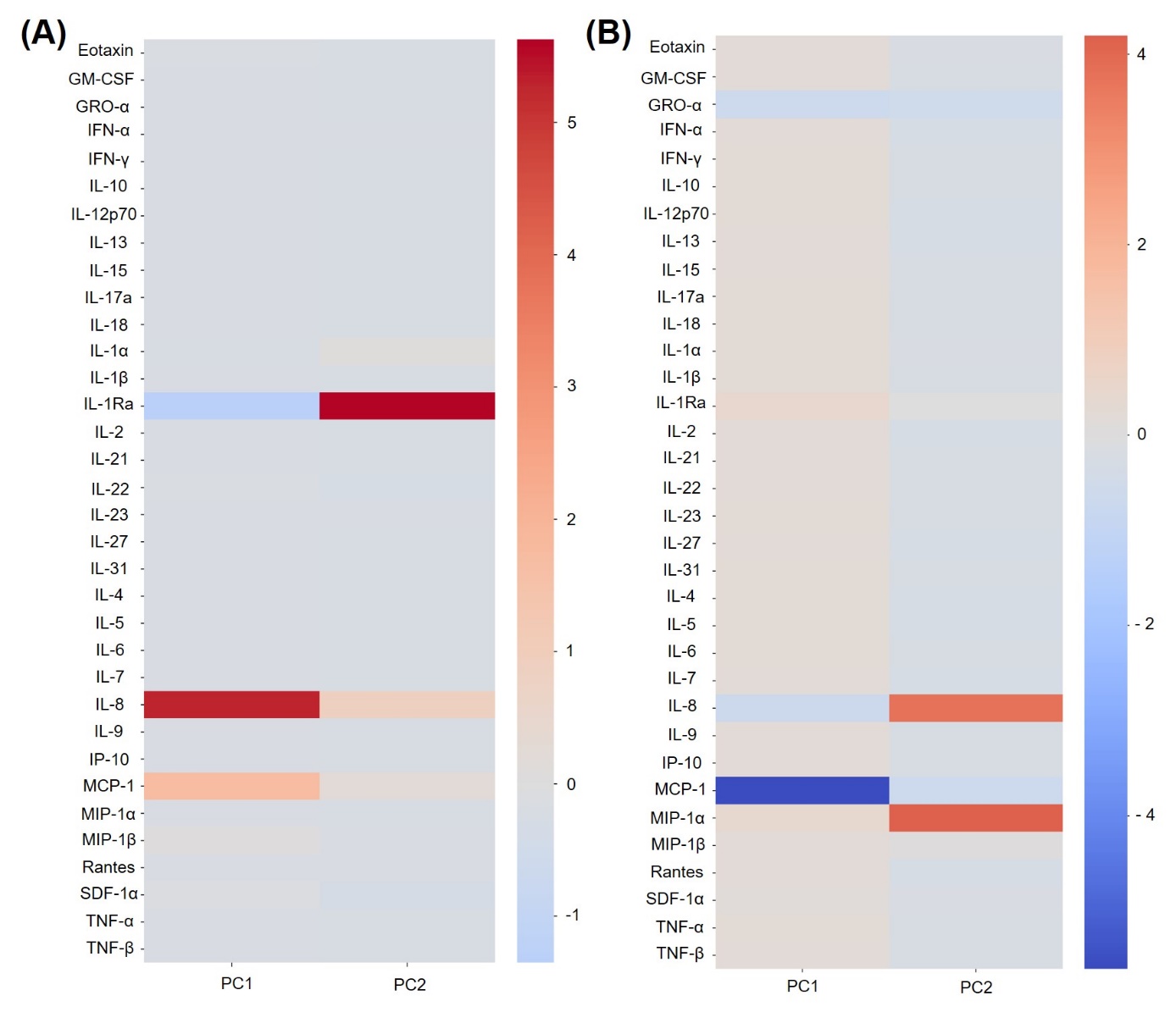


Figure S5. Cytokines and chemokines variance contribution to principal components 1 and 2 (PC1-2): (A) Variance contribution to the EoC model. (B) Variance contribution to the FT model.
